# Caboxamycin biosynthesis pathway and identification of novel benzoxazoles produced by cross‐talk in *Streptomyces* sp. NTK 937

**DOI:** 10.1111/1751-7915.12716

**Published:** 2017-04-18

**Authors:** Armando A. Losada, Carolina Cano‐Prieto, Raúl García‐Salcedo, Alfredo F. Braña, Carmen Méndez, José A. Salas, Carlos Olano

**Affiliations:** ^1^ Departamento de Biología Funcional e Instituto Universitario de Oncología del Principado de Asturias (I.U.O.P.A)Universidad de Oviedo33006OviedoSpain

## Abstract

*Streptomyces* sp. NTK937, producer of benzoxazole antibiotic caboxamycin, produces in addition a methyl ester derivative, *O*‐methylcaboxamycin. Caboxamycin cluster, comprising one regulatory and nine structural genes, has been delimited, and each gene has been individually inactivated to demonstrate its role in the biosynthetic process. The *O*‐methyltransferase potentially responsible for *O*‐methylcaboxamycin synthesis would reside outside this cluster. Five of the genes, *cbxR, cbxA, cbxB, cbxD* and *cbxE*, encoding a SARP transcriptional regulator, salicylate synthase, 3‐oxoacyl‐ACP‐synthase, ACP and amidohydrolase, respectively, have been found to be essential for caboxamycin biosynthesis. The remaining five structural genes were found to have paralogues distributed throughout the genome, capable of partaking in the process when their cluster homologue is inactivated. Two of such paralogues, *cbxC’* and *cbxI’*, coding an AMP‐dependent synthetase‐ligase and an anthranilate synthase, respectively, have been identified. However, the other three genes might simultaneously have more than one paralogue, given that *cbxF* (DAHP synthase), *cbxG* (2,3‐dihydro‐2,3‐dihydroxybenzoate dehydrogenase) and *cbxH* (isochorismatase) have three, three and five putative paralogue genes, respectively, of similar function within the genome. As a result of genetic manipulation, a novel benzoxazole (3′‐hydroxycaboxamycin) has been identified in the salicylate synthase‐deficient mutant strain ΔcbxA. 3′‐hydroxycaboxamycin derives from the cross‐talk between the caboxamycin and enterobactin pathways.

## Introduction

**Figure 1:**
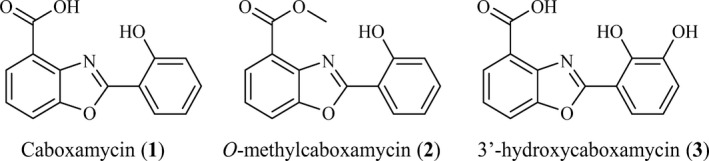
Chemical structures of caboxamycin and caboxamycin derivatives identified in this work.

*Streptomyces* sp. NTK937, a deep‐sea sediment actinomycete collected off the coast of the Canary Islands, produces the benzoxazole antibiotic caboxamycin (**1**) (Fig. 1) (Hohmann *et al*., 2009). The benzoxazole family of compounds include members with two benzoxazole motifs such as UK‐1 (Ueki *et al*., 1993), AJI9561 (Sato *et al*., 2001) or nataxazole (Sommer *et al*., 2008), and members such as caboxamycin and A33853 (Michel *et al*., 1984) with a simpler structure, bearing only one benzoxazole motif, formed by the fusion of a 3‐hydroxyanthranilate and a salicylate moieties or two moieties of 3‐hydroxyanthranilate respectively. Even though caboxamycin (**1**) has a lower cytotoxicity than UK‐1 or nataxazole, it has shown additional antibiotic properties against Gram‐positive bacteria, as well as inhibition of phosphodiesterases, potential targets for treating asthmatic inflammation and chronic obstructive pulmonary disease (Hohmann *et al*., 2009). On the other hand, the chemically obtained caboxamycin methyl ester (**2**) (Fig. 1) has been recently reported to inhibit hepatitis C virus replication (Talley *et al*., 2016). These activities are a small part of the wide array of pharmacological activities shown by benzoxazole scaffold‐carrying molecules (Singh *et al*., 2015). Therefore, elucidation of caboxamycin biosynthetic cluster would be of great interest to study the many possibilities benzoxazoles can offer. The knowledge gathered by unravelling the caboxamycin pathway will open up the opportunity to generate novel benzoxazole derivatives with improved biological properties such as antibiotic, antifungal, cytotoxic and others. This aim might be fulfilled using different biotechnological approaches including combinatorial biosynthesis and mutasynthesis.

In a previous work, we have reported the genome sequencing and analysis of *Streptomyces* sp. NTK937 (Olano *et al*., 2014), identifying up to 35 putative gene clusters probably involved in secondary metabolite production. Caboxamycin biosynthesis might be the result of the fusion of a 3‐hydroxyanthranilic acid (3HAA) unit with a salicylic acid (SA) moiety. The most reasonable biosynthetic origin of 3HAA might be the same as in the calcimycin, nataxazole and A33853 biosynthesis pathways, which have been previously reported (Wu *et al*., 2011; Cano‐Prieto *et al*., 2015a; Lv *et al*., 2015), that is, as a direct derivative of chorismate instead of a catabolic by‐product of tryptophan. Meanwhile, biosynthesis of SA has been described in two possible ways, both stemming from chorismate, either in two steps via isochorismate synthase and isochorismate‐pyruvate lyase (Gross and Loper, 2009), or in a single step performed by a salicylate synthase (Kerbarh *et al*., 2005). These putative biosynthetic origins of caboxamycin can be used to identify the correct biosynthesis gene cluster. Additionally, the *Streptomyces* sp. NTK937 genome contains several non‐ribosomal peptide synthase (NRPS)‐harbouring clusters (Olano *et al*., 2014), one of which, based on its similarity to the enterobactin cluster in *Streptomyces* sp. Tü6176 (Cano‐Prieto *et al*., 2015b), might be responsible for the production of this siderophore. The presence of this cluster in caboxamycin producer *Streptomyces* sp. NTK937 led us to investigate a possible relationship between caboxamycin and enterobactin biosynthesis pathways through the shikimate pathway, the source of their common precursor, chorismate. This type of metabolic relationship was previously reported for nataxazole and enterobactin pathways (Cano‐Prieto *et al*., 2015b).

In this work, we report the identification and characterization of the complete gene cluster for caboxamycin biosynthesis in *Streptomyces* sp. NTK937. Individual and independent inactivation of all the genes in the cluster and the heterologous expression of the cluster led us to propose a plausible pathway for caboxamycin biosynthesis and to identify several paralogues distributed throughout *Streptomyces* sp. NTK937 genome that collaborate in the process. Complementation of the caboxamycin defective mutant strains generated in this work using orthologue genes involved in the biosynthesis of nataxazole led to further understanding of the nataxazole biosynthesis pathway. Furthermore, we show the production of two caboxamycin derivatives, one derived of an *O*‐methyltransferase activity encoded by a gene residing outside of the cluster, and a second one generated by cross‐talk between caboxamycin and enterobactin biosynthesis pathways.

## Results

### Identification of caboxamycin biosynthesis gene cluster

**Figure 2:**
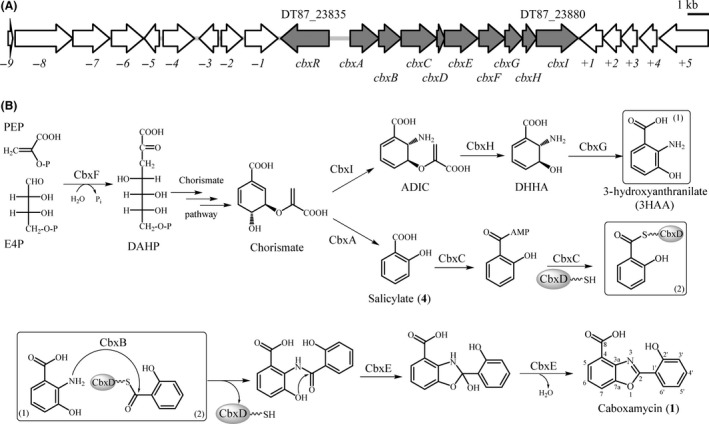
(A) Genetic organization of caboxamycin biosynthesis gene cluster in *Streptomyces* sp. NTK937. (B) Proposed pathway for caboxamycin biosynthesis. 3HAA, 3‐hydroxyanthranilate; ADIC, 2‐amino‐2‐deoxyisochorismate; DAHP, 3‐deoxy‐D‐arabinohept‐2‐ulosonate‐7‐phosphate; DHHA,* Trans*‐2,3‐dihydro‐3‐hydroxyanthranilate; E4P, erythrose‐4‐phosphate; PEP, phosphoenolpyruvate.

Having considered the possible biosynthetic origin of caboxamycin as a fusion of 3HAA and SA, the sequenced genome of *Streptomyces* sp. NTK937 (accession no. JJOB01000000) was scanned for putative adequate open reading frames. For the origin of 3HAA, the hypothesis is analogue to that of closely related benzoxazole nataxazole (Cano‐Prieto *et al*., 2015a), where it derives from modification of chorismate, as opposed to catabolism of tryptophan by modification of kynurenine (Li *et al*., 2009). Four anthranilate synthase coding genes were identified, two of which (DT87_23880 and DT87_29875) were located next to additional genes encoding the required enzyme activities 2,3‐dihydro‐2,3‐dihydroxybenzoate dehydrogenase (DT87_23870 and DT87_28500) and isochorismatase (DT87_23875 and DT87_29880) for the biosynthesis of 3HAA, as well as a DAHP synthase (DT87_23865 and DT87_29890) that would favour the biosynthesis of the chorismate precursor 3‐deoxy‐D‐arabinohept‐2‐ulosonate‐7‐phosphate (DAHP) (Tables 1 and 2). For SA, two possible biosynthetic pathways were evaluated: a direct conversion from chorismate performed by a salicylate synthase as described for Irp9 in yersiniabactin biosynthesis (Kerbarh *et al*., 2005), a situation that has been also noted in the cross‐talk production of UK‐1 by nataxazole producer *Streptomyces* sp. Tü6176 (Cano‐Prieto *et al*., 2015b); or through a two‐step process performed by isochorismate synthase and isochorismate‐pyruvate lyase, such as the pyochelin PchA‐PchB system (Gross and Loper, 2009). Genome mining revealed only one isochorismate synthase (DT87_28495, *entC*) (Table 2), but no presence of a putative isochorismate‐pyruvate lyase was detected. However, two putative salicylate synthases were identified. One of the salicylate synthase coding genes, DT87_25870 (Table 2), contains a frameshift mutation that would cause the putative protein to become non‐functional, as was demonstrated by heterologous expression of the gene in *S. albus* J1074, where no SA production was observed (Supporting information, Fig. S1). The second salicylate synthase coding gene, DT87_23840, was shown to direct the biosynthesis of SA when expressed in *S. albus* J1074 (Supporting information, Fig. S1), and lies in close vicinity to one of the DAHP synthase, 2,3‐dihydro‐2,3‐dihydroxybenzoate dehydrogenase, isochorismatase and anthranilate synthase clusters (DT87_23865 to DT87_23880) previously mentioned (Fig. 2A and Table 1). The whole set of genes was recognized by antiSMASH analysis, which includes the putative genes as part of a larger NRPS‐containing biosynthetic cluster. Taking in consideration the genes located in DT87_23835 to DT87_23880 region, which encode all the activities that might be required for the biosynthesis of caboxamycin, we propose a putative pathway for the biosynthesis of this benzoxazole (Fig. 2B).

**Table 1:** Caboxamycin biosynthesis gene cluster region in *Streptomyces* sp. NTK937

Gene	Location DT87_	Proposed function	Orthologue in nataxazole cluster^a^	Most similar protein^b^
*orf‐9*	23 790	Acyl carrier protein	–	WP_043498560 (74/86) *S. glaucescens*
*orf‐8*	23 795	Class III aminotransferase	–	WP_052413620 (81/88) *S. glaucescens*
*orf‐7*	23 800	AMP‐dependent synthetase‐ligase	–	WP_051422268 (99/99) *Streptomyces* sp. DpondAA‐B6
*orf‐6*	23 805	Phenylalanine‐specific permease	–	WP_051422266 (99/99) *Streptomyces* sp. DpondAA‐B6
*orf‐5*	23 810	TetR‐family transcriptional regulator	–	WP_043498549 (78/86) *S. glaucescens*
*orf‐4*	23 815	EmrB/QacA drug resistance transporter	–	WP_052414047 (78/86) *S. glaucescens*
*orf‐3*	23 820	Leucine‐carboxyl methyltransferase	–	WP_043483949 (60/68) *S. olivaceus*
*orf‐2*	23 825	AsnC‐family transcriptional regulator	–	WP_052410747 (76/86) *S. olivaceus*
*orf‐1*	23 830	EmrB/QacA drug resistance transporter	–	WP_037748866 (99/99) *Streptomyces* sp. DpondAA‐B6
*cbxR*	23 835	SARP‐family transcriptional regulator	*natR4* (51.7/39.3)	WP_052410743 (99/99) *S. olivaceus*
*cbxA*	23 840	Salicylate synthase	CF54_20720^c^(60.7/49.3)	WP_028441039 (99/100) *Streptomyces* sp. DpondAA‐B6
*cbxB*	23 845	3‐oxoacyl‐ACP synthase III	*natS* (66.8/54.8)	WP_031037807 (62/76) *S. olivaceus*
*cbxC*	23 850	AMP‐dependent synthetase‐ligase	*natL1* (70.6/60.8)	WP_038519516 (65/74) *Amycolatopsis japonica*
			*natL2* (48.2/22.0)	
*cbxD*	23 855	Acyl carrier protein	*natAC1* (60.3/46.5)	SBU95399 *Streptomyces* sp. OspMP‐M45
			*natAC2* (49.6/22.6)	
*cbxE*	23 860	Amidohydrolase	*natAM* (67.4/55.6)	WP_031508998 (62/75) *S. megasporus*
*cbxF*	23 865	Class II DAHP synthase	*natAL* (60.3/48.0)	SCD89909 (99/99) *Streptomyces* sp. PalvLS‐984
*cbxG*	23 870	2,3‐dihydro‐2,3‐ dihydroxybenzoate dehydrogenase	*natDB* (70.9/62.0)	SCD8992 (98/98) *Streptomyces* sp. PalvLS‐984
*cbxH*	23 875	Isochorismatase	*natIS* (69.5/63.4)	EsmA4 (72/78) *S. antibioticus*
*cbxI*	23 880	Anthranilate synthase	*natAN* (65.9/54.5)	WP_051422252 (99/99) *Streptomyces* sp. DpondAA‐B6
*orf+1*	23 885	ATP‐binding ABC transporter	–	WP_030870615 *S. violaceoruber* (89/94)
*orf+2*	23 890	Molybdate ABC transporter	–	WP_028441031 (100/100) *Streptomyces* sp. DpondAA‐B6
*orf+3*	23 895	Molybdate binding protein	–	WP_028441030 (99/98) *Streptomyces* sp. DpondAA‐B6
*orf+4*	23 900	MerR‐family transcriptional regulator	–	WP_003992129 (91/96) *S. viridochromogenes*
*orf+5*	23 910	Cobalamin‐independent methionine synthase	–	WP_028441028 (99/99) *Streptomyces* sp. DpondAA‐B6

**a**. % level of identity at gene and deduced protein in parenthesis.

**b**.% level of identity and similarity in parenthesis.

**c**. Involved in UK‐1 biosynthesis in nataxazole producer *Streptomyces* sp. Tü6176.

**Table 2:** Caboxamycin biosynthesis paralogue genes located in *Streptomyces* sp. NTK937

Gene	Location DT87_	Proposed function	Most similar protein^a^
*cbxA’*	25 870	Salicylate synthase	WP_028443031 (97/97) *Streptomyces* sp. DpondAA‐B6
*cbxC’*	23 710	AMP‐dependent synthetase‐ligase	WP_028441054 (99/99) *Streptomyces* sp. DpondAA‐B6
*cbxI’*	29 875	Anthranilate synthase	WP_051422472 (99/99) *Streptomyces* sp. DpondAA‐B6
*cbxH’*	29 880	Isochorismatase	GAT84341 (79/88) *Streptomyces* sp. F‐3
*cbxG’*	29 885	2,3‐dihydro‐2,3‐ dihydroxybenzoate dehydrogenase	WP_028443631 (98/99) *Streptomyces* sp. DpondAA‐B6
*cbxF’*	29 890	Class II DAHP synthase	WP_028443630 (99/99) *Streptomyces* sp. DpondAA‐B6
*cbxF’’*	05 590	Class II DAHP synthase	WP_031081129 (97/99) *Streptomyces* sp. NRRL WC‐3549
*entA*	28 500	2,3‐dihydro‐2,3‐ dihydroxybenzoate dehydrogenase; enterobactin biosynthesis gene cluster	WP_028440609 (98/97) *Streptomyces* sp. DpondAA‐B6
*entB*	28 485	Isochorismatase; enterobactin biosynthesis gene cluster	WP_014049800 (92/91) *Streptomyces* sp. SirexAA‐E
*entC*	28 495	Isochorismate synthase; enterobactin biosynthesis gene cluster	WP_028440610 (99/99) *Streptomyces* sp. DpondAA‐B6

a % identity/similarity in parenthesis

Bioinformatic analysis of the putative cluster gives some interesting homologies with previously described enzymes that concur with our general hypothesis, such as the salicylate synthase CbxA being most similar to salicylate synthases SsfH of the SF2575 (Pickens *et al*., 2010) and Ccb3 of the celesticetin (Janata *et al*., 2015) clusters, or the anthranilate synthase CbxI being most similar to phenazine biosynthesis PhzE, which has actually been described as member of a very small family of ADIC synthases (Culbertson and Toney, 2013) instead of a true anthranilate synthase. Meanwhile, others share very diverse homologies, such as the AMP‐dependent synthetase‐ligase CbxC that shares homologies with a series of adenylating enzymes such as EsmD2, SnbA, MxcE and TrsI tha participate in the biosynthesis of saphenamycin (Rui *et al*., 2012) pristinamycin (De Crécy‐Lagard *et al*., 1997), myxochelin (Gaitatzis *et al*., 2001) and triostin A (Praseuth *et al*., 2008), respectively, and whose only common characteristic is the adenylation of a 2‐hydroxy‐aromatic acid, all of them different from salicylic acid.

### Genetic characterization of the caboxamycin biosynthesis gene cluster in Streptomyces sp. NTK937

**Figure 3:**
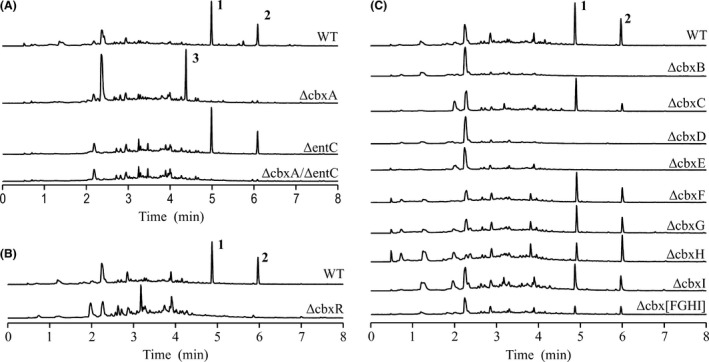
(A) UPLC analysis of *Streptomyces* sp. NTK937 wild‐type (WT) and mutant strains ΔcbxA, ΔentC and ΔcbxA/ΔentC. (B) UPLC analysis mutant strain ΔcbxR. (C) UPLC analysis of mutant strains carrying individual inactivation of each structural gene of the caboxamycin biosynthesis cluster. Extracts were obtained from cultures grown in R5A medium for 7 days. UPLC chromatograms were analysed at 330 nm. Labelled peaks correspond: caboxamycin A (**1**), *O*‐methylcaboxamycin (**2**), 3′‐hydroxycaboxamycin (**3**).

The involvement of the genes located in DT87_23835 to DT87_23880 region in caboxamycin biosynthesis was initially proven through inactivation of the salicylate synthase coding gene DT87_23840 (*cbxA*), which led to mutant strain ΔcbxA. Analysis of products accumulated by mutant ΔcbxA in comparison with the wild‐type strain (Fig. 3A) showed the disappearance of two peaks, one corresponding to caboxamycin (**1**) and a second one (**2**) with characteristic benzoxazole absorption spectrum, UPLC retention time of 6.0 min and mass of *m/z* 270 [*M*+H]^+^. Structural elucidation of compound **2** showed it corresponds to benzoxazole *O*‐methylcaboxamycin (Fig. 1), compound previously generated only by total synthesis (Talley *et al*., 2016). In addition to the disappearance of caboxamycin (**1**) and *O*‐methylcaboxamycin (**2**), mutant strain ΔcbxA accumulated a novel compound (**3**) with an absorption spectrum reminiscent of benzoxazoles, UPLC retention time of 4.3 min and mass of *m/z* 272 [*M*+H]^+^. Structural elucidation of compound **3** showed it corresponds to 3′‐hydroxycaboxamycin (Fig. 1), which includes a 2,3‐dihydroxybenzoate moiety, probably originated from the enterobactin biosynthesis pathway, cluster where the aforementioned isochorismate synthase DT87_28495 (*entC*) lies (Table 2). The existence of a cross‐talk between caboxamycin and enterobactin biosynthesis pathways that led to the production of 3′‐hydroxycaboxamycin (**3**) in mutant strain ΔcbxA was demonstrated through inactivation of the enterobactin isochorismate synthase coding gene *entC* both in *Streptomyces* sp. NTK937 and in ΔcbxA strain, leading to mutant strains ΔentC and ΔcbxA/ΔentC respectively. Analysis of products accumulated by mutant ΔentC showed the normal production of caboxamycin (**1**) and *O*‐methylcaboxamycin (**2**) while the double mutant strain ΔcbxA/ΔentC was unable to produce any benzoxazole, including 3′‐hydroxycaboxamycin (**3**) (Fig. 3A).

According to our biosynthetic hypothesis, the caboxamycin biosynthesis gene cluster might require only the nine distinct open reading frames lying between salicylate synthase and anthranilate synthase (DT87_23840 to DT87_23880, *cbxA* to *cbxI*), and an opposite direction gene upstream of salicylate synthase *cbxA* that codifies a SARP‐like regulator (DT87_23835, *cbxR*). Indeed, the inactivation of *cbxR* leading to mutant strain ΔcbxR abolishes benzoxazole production (Fig. 3B), result that justifies its possible role as a positive transcriptional regulator.

In addition to *cbxR* and *cbxA*, each gene in the caboxamycin biosynthesis cluster was independently inactivated leading to the corresponding mutant strains (Fig. 3C). As it was observed in the aforementioned mutant strains ΔcbxA and ΔcbxR, production of **1** and **2** was suppressed in ΔcbxB, ΔcbxD and ΔcbxE mutant strains. The remaining mutant strains, ΔcbxC, ΔcbxF, ΔcbxG, ΔcbxH and ΔcbxI, showed normal production of caboxamycin (**1**) with minor alterations in the production of *O*‐methylcaboxamycin (**2**). Even the simultaneous deletion of all four genes involved in the biosynthesis of precursor 3HAA (*cbxF*,* cbxG*,* cbxH* and *cbxI*), leading to mutant strain Δcbx[FGHI], could only reduce the production of caboxamycin by approximately two‐thirds (Fig. 3C). These results are in apparent contradiction with our initial pathway proposal, as all the genes present in the cluster were considered essential in caboxamycin biosynthesis, and point to additional genes in the genome of *Streptomyces* sp. NTK937 being involved in caboxamycin biosynthesis (see below).

On the other hand, simultaneous inactivation by gene replacement of several contiguous genes both upstream of *cbxR* (*orf‐3*,* orf‐2* and *orf‐1*) and downstream of *cbxI* (*orf+1*,* orf+2* and *orf+3*) showed no effect on caboxamycin production, and neither did the individual inactivation of more distant putative regulators of the TetR and MerR families of transcriptional regulators (*orf‐5* and *orf+4*) (Supporting information, Fig. S2), thus delimiting the borders of the caboxamycin biosynthesis gene cluster from *cbxR* to *cbxI*.

### Heterologous expression of the caboxamycin biosynthesis gene cluster

**Figure 4:**
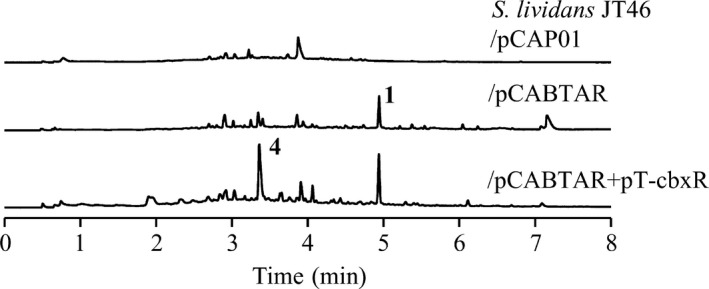
UPLC analysis showing production of caboxamycin (**1**) and salicylic acid (**4**) in cultures of *S. lividans* JT46 carrying pCAP01, pCABTAR or pCABTAR + pT‐cbxR, grown in R5A medium for 7 days. UPLC chromatograms were analysed at 330 nm.

The limits of the cluster were further established through heterologous expression of the cluster using a TAR‐cloning approach. The nine structural genes transcribed in the same direction, *cbxABCDEFGHI*, were expressed in *Streptomyces lividans* JT46 host using pCABTAR and leading to a noticeable production of caboxamycin (**1**). This production was increased by simultaneous overexpression of the SARP regulator coding gene *cbxR* (pT‐cbxR), not originally included in pCABTAR (Fig. 4). Additionally, the simultaneous expression of *cbxABCDEFGHI* and *cbxR* resulted in a remarkable accumulation of the precursor SA (**4**) together with the final product caboxamycin (Fig. 4). In none of these heterologous expression approaches, the production of *O*‐methylcaboxamycin (**2**) was detected, due to the absence of a specific *O*‐methyltransferase among the structural genes of caboxamycin biosynthesis harboured in pCABTAR.

### Genetic characterization of the caboxamycin biosynthesis gene cluster paralogues in Streptomyces sp. NTK937

**Figure 5:**
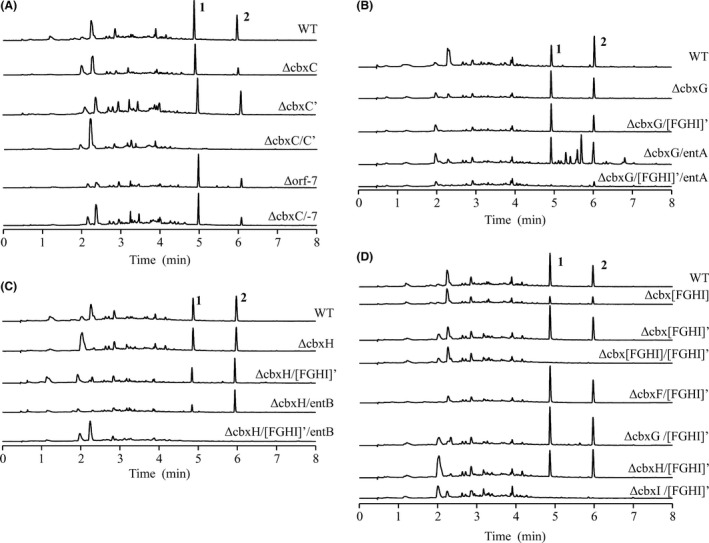
UPLC analysis of *Streptomyces* sp. NTK937 wild‐type (WT) and different mutant strains. (A) AMP‐dependent synthetase‐ligase‐deficient mutant strains. (B) 2,3‐dihydro‐2,3‐dihydroxybenzoate dehydrogenase‐deficient mutant strains. (C) Isochorismatase‐deficient mutant strains. (D) Anthranilate synthase‐deficient mutant strains. Extracts were obtained from cultures grown in R5A medium for 7 days. UPLC chromatograms were analysed at 330 nm. Labelled peaks correspond to caboxamycin A (**1**), *O*‐methylcaboxamycin (**2**) and 3′‐hydroxycaboxamycin (**3**).

We attempted to clarify the question of the presence of additional genes in the genome of *Streptomyces* sp. NTK937 that complement the loss activity of caboxamycin biosynthesis genes *cbxCFGHI* when any of these was inactivated. As it has been mentioned above, several paralogues of identical putative function as those present within the caboxamycin biosynthesis cluster have been identified throughout the genome of *Streptomyces* sp. NTK937, and they could act as back‐up copies supplying the lost function in the genes replacement mutants. In the case of *cbxC,* multiple possibilities for AMP‐dependent synthetase‐ligase coding genes exist within the genome. One of such genes is DT87_23710 (*cbxC’*), located only 24 *orfs* upstream from the beginning of the caboxamycin cluster, whose deduced product presents an amino acid sequence identity of 51% to CbxC. The double mutant strain ΔcbxC/C’ showed no production of caboxamycin nor *O*‐methylcaboxamycin, while the mutant strain ΔcbxC’ still produced normal amounts of both compounds (Fig. 5A). These results clearly demonstrate the role of *cbxC’* as a back‐up system for caboxamycin biosynthesis. Yet another AMP‐dependent synthetase‐ligase coding gene, *orf‐7* (DT87_23800), exists in between *cbxC* and *cbxC’*, only 7 *orfs* upstream from *cbxR* (Fig. 2A). However, the inactivation of orf‐7 independently (Δorf‐7) or in a double mutant (ΔcbxC/‐7) had no effect over the biosynthesis of benzoxazoles (Fig. 5A), therefore excluding *orf‐7* from any role in caboxamycin biosynthesis. This is coincident with the bioinformatic analysis of *orf‐7* deduced product, which shows higher identity between CbxC and CbxC’ than between CbxC and Orf‐7 (16%).

After failing to cancel out caboxamycin production in the ΔcbxF mutant, DAHP synthase paralogues, *cbxF*,* cbxF’* (DT87_29890) and *cbxF’’* (DT87_05590), were analysed by sequence homology, together with their orthologues from nataxazol producer *Streptomyces* sp. Tü6176, *natAL* and CF54_24340 (located outside nataxazole biosynthetic cluster). Protein sequence alignments show that CbxF presents higher identity to CbxF’ (62%) and NatAL (46%) than to CbxF’’ (42%). Besides, CbxF’’ is 91% identical to CF54_24340. Given that DAHP synthase is an enzyme required for primary metabolism as the first dedicated step in the chorismate pathway, CF54_24340 and *cbxF’’* might fulfil this role in their respective strains, which leaves out *cbxF’* as the putative paralogue of *cbxF* for caboxamycin biosynthesis. Nevertheless, a double mutant strain ΔcbxF/F’ is still able to synthesize **1** and **2**, and so is the alternative ΔcbxF/F’’ (Supporting information, Fig. S12A). The generation of a triple ΔcbxF/F’**/**F’’ is unfeasible, given that at least one DAHP synthase is required to maintain the vital shikimate pathway. We sought to repress the action of whichever aldolase is responsible for primary metabolism by supplementing culture media with each individual aromatic aminoacid (Light and Anderson, 2013), but already in the wild‐type strain is shown that all three aldolases present in the genome are repressed by a tryptophan feedback, annulling the biosynthesis of benzoxazoles (Supporting information, Fig. S12B). Therefore, it is impossible to ascertain whether all three aldolases are simultaneously partaking in benzoxazole biosynthesis, or whether the third one only comes into action when the other two are absent.

In the case of 2,3‐dihydro‐2,3‐dihydroxybenzoate dehydrogenase (*cbxG*) and isochorismatase (*cbxH*), three and five paralogues exist, respectively, for each of them within the genome, with the *cbxG* paralogue lying always in close vicinity to a *cbxH*‐like gene. After individual ΔcbxG and ΔcbxH mutants failed to suppress caboxamycin production, we took into consideration the other two genomic loci where they are closely related, one of which is next to the aforementioned *cbxF’* and the other one being within the enterobactin cluster (*entA* and *entB*), that has already been proven to participate in a cross‐talk with the caboxamycin pathway for the biosynthesis of 3′‐hydroxycaboxamycin in ΔcbxA mutant strain. The generation of double mutants in any of these genes in tandem with their caboxamycin biosynthesis gene paralogue failed to abrogate caboxamycin biosynthesis, but the simultaneous replacement of all three genes in ΔcbxG/[FGHI]’/entA and ΔcbxH/[FGHI]’/entB mutants succeeded in abolishing caboxamycin and *O*‐methylcaboxamycin biosynthesis (Fig. 5B and C).

Finally, anthranilate synthase (*cbxI*) counts up to four putative paralogues within the *Streptomyces* sp. NTK937 genome. After showing that a mutant strain with all four 3HAA‐related genes removed (Δcbx[FGHI]) was still able to synthesize caboxamycin (Fig. 3C), although at lower production levels, we succeeded in making a non‐producing mutant by deleting in tandem the previously mentioned locus *cbx[FGHI]’*. Given that there are four gene products in this group, *cbxF’*,* cbxG’*,* cbxH’* and *cbxI’* that could potentially partake in caboxamycin biosynthesis, it became necessary to replace these genes (*cbx[FGHI]’*) in each corresponding individual caboxamycin biosynthesis gene‐deleted mutant (ΔcbxF, ΔcbxG, ΔcbxH, ΔcbxI). Only the ΔcbxI/[FGHI]’ mutant resulted in a non‐producing phenotype (Fig. 5D), given that the others are still supported, respectively, by the actions of *cbxF’’*,* entA* and *entB*, therefore indicating that the sole real paralogue of *cbxI* would be *cbxI’* (DT87_29875).

### Cross‐complementation of caboxamycin non‐producer mutants using nataxazole biosynthesis genes from Streptomyces sp Tü6176

The reintroduction of each replaced gene succeeded in restoring the biosynthesis of caboxamycin (**1**) and/or *O*‐methylcaboxamycin (**2**) in all non‐producing mutants, whether simple, double or triple (Supporting information, Fig. S3A to S11). An exception is the salicylate synthase DT87_25870 (cbxA’), which, as expected due to the presence of a frameshift mutation, did not restore caboxamycin production (Supporting information, Fig. S4), and its heterologous expression in *S. albus* did not induce SA accumulation, unlike its homologues (Supporting information, Fig. S1).

Meanwhile, expression of nataxazole biosynthesis cluster orthologue genes in these same mutant strains was successful in most cases, although often with a lower production (Supporting information, Fig. S3A, S5 to S7 and S9 to S11), with notable exception of *natAM* which failed to complement the ΔcbxE mutant strains, in which, on the other hand, the biosynthesis of caboxamycin was only restored to minimal levels by complementation with the original *cbxE* (Supporting information, Fig. S8). In the case of CF54_20720, which encodes a salicylate synthase responsible for UK‐1 biosynthesis in *Streptomyces* sp. Tü6176, the complementation of ΔcbxA is partial, leading to simultaneous production of **1**,** 2** and **3** (Supporting information, Fig. S4), the latter being the natural cross‐talk product of the ΔcbxA mutant strain. Furthermore, the fact that *natL1*/*natAC1* can, respectively, restore **1** and **2** production while *natL2*/*natAC2* cannot (Supporting information, Fig. S4 and S5) gives further proof on their performing order in nataxazole biosynthesis pathway, making *natL1/natAC1* likely responsible for the 6MSA‐3HAA fusion, as they are capable of performing this fusion with the closely related SA, while *natL2/natAC2* would be in charge of the 3HAA‐3HAA benzoxazole scaffold formation. Coincidentally, the *natL2*‐overexpressing ΔcbxC/ΔcbxC’ mutant strain shows the production of 3′‐hydroxy‐caboxamycin (**3**) (Supporting information, Fig. S6) which further indicates NatL2 preference for binding 3HAA‐like compounds such as 2,3‐dihydroxybenzoate, which only differs in switching the amino group for a hydroxyl group.

Ectopic expression of the *cbxR* regulator in *Streptomyces* sp. NTK937 wild‐type strain resulted in yields up to 40 times higher for **1** and **2** (Supporting information, Fig. S3B), while its nataxazole orthologue, *natR4*, although capable of complementing the ΔcbxR mutant (Supporting information, Fig. S3A), has barely any effect on the wild‐type strain caboxamycin production levels. The nataxazole biosynthesis cluster repressors, belonging to the TetR‐family of transcriptional regulators, *natR2* and *natR3*, were likewise ectopically expressed in *Streptomyces* sp. NTK937, but they failed to exert any decrease in caboxamycin production, in comparison with their role in nataxazole biosynthesis (Supporting information, Fig. S3B).

### Biological activity of caboxamycin and derived benzoxazoles

The antibiotic activities of caboxamycin (**1**), *O*‐methylcaboxamycin (**2**) and 3′‐hydroxycaboxamycin (**3**) were tested by antibiotic disc diffusion assays against *S. albus* J1074, *E. coli*,* Staphylococcus aureus, Micrococcus luteus* and the yeast *Candida albicans*. None of them showed activity against bacteria, but growth of *C. albicans* was inhibited by 2.5 μg of caboxamycin or 10 μg of 3′‐hydroxycaboxamycin. These results, in particular the absence of antibiotic activity against Gram‐positive bacteria, are puzzling considering that previous reports showed caboxamycin being active against *Bacillus subtilis*,* Staphylococcus lentus* and *Staphylococcus epidermidis* (Hohmann *et al*., 2009). However, after running the biological activity tests several times, we can conclude that *S. albus*,* S. aureus* and *M. luteus* are clearly not affected, in our experimental conditions, by caboxamycin or its two derivatives.

Cytotoxicity of **1** – **3** was tested against a selection of tumoral cell lines: HT29 (colon), A549 (lung), MDA‐MB‐231 (breast), AGS (gastric) and A2780 (ovarian), as well as a mouse non‐malignant cell line NIH/3T3 as control test. Only **2** showed activity at IC_50_ concentrations of 3.7 μM (HT29), 2.7 μM (A549), 6.1 μM (MDA‐MB‐231), 5 μM (AGS), 4.93 μM (A2780) and 5.14 μM (NIH/3T3), while **1** and **3** showed no activity up to 10 μM. In contrast to the relative activities of AJ9561 and nataxazole, where the presence of a methyl ester group in nataxazole renders this compound less active than AJ9561 (Cano‐Prieto *et al*., 2015a), the methyl ester group in *O*‐methylcaboxamycin confers to this compound a higher cytotoxic activity than that of caboxamycin.

## Discussion

The *Streptomyces* sp. NTK937 caboxamycin biosynthetic cluster was identified *in silico* and confirmed by both gene inactivation and heterologous expression in *S. lividans* JT46. It consists of ten genes, nine of which encode structural enzymes responsible for biosynthesis, plus one SARP‐like transcriptional regulator. These genes might be organized in as many as four distinct operons: one for the regulator, and the others organized as *cbxABCDE*,* cbxFG* and *cbxHI*, given the fact of their overlapping start and stop codons. The caboxamycin pathway involves the synthesis of 3‐hydroxyanthranilic and salicylic acid from chorismate, their activation and attachment into an ACP and their subsequent condensation involving the participation of amidohydrolase CbxE. The *O*‐methyltransferase required for *O*‐methylcaboxamycin biosynthesis is not located in the vicinity of the cluster and thus has yet to be identified. This enzymatic activity, in contrast to what has been shown for nataxazole biosynthesis in *Streptomyces* sp. Tü6176 (Cano‐Prieto *et al*., 2015a), does not appear to constitute a resistance mechanism as caboxamycin is not active against *Streptomyces* spp.

While a structural gene lying outside the cluster is uncommon for actinomycetes, several cases have been reported (Karki *et al*., 2010; Huang *et al*., 2015), including the closely related nataxazole cluster, where an undetermined *O*‐methyltransferase outside the cluster is responsible for the conversion of AJI9561 into nataxazole (Cano‐Prieto *et al*., 2015a). Even less common is the presence of several paralogues codifying genes that can act as backups for the cluster structural genes (Tahlan *et al*., 2004; Engelhardt *et al*., 2010). In the case of AMP‐dependent synthetase‐ligase coding *cbxC’* and the paralogue set *cbx(FGHI)’,* they could be considered as isoenzymes, as they are not ascribed to any other biosynthetic pathway. On the other hand, in the case of 2,3‐dihydro‐2,3‐dihydroxybenzoate dehydrogenase *entA* and isochorismatase *entB,* they have been demonstrated to exhibit a certain degree of catalytic promiscuity, given that they accept 3‐hydroxyanthranilate precursors as well as their expected 2,3‐dihydroxybenzoate precursors, as they can back up the loss of *cbxG* and *cbxH* respectively. Besides, isochorismate synthase EntC show a cross‐talk interaction with the caboxamycin biosynthesis pathway when salicylate synthase coding *cbxA* is removed, leading to the generation of a novel caboxamycin derivative: 3′‐hydroxycaboxamycin.

Finally, most of the caboxamycin biosynthesis genes, including the regulatory gene *cbxR*, can be substituted by their corresponding orthologue from the nataxazole biosynthesis gene cluster. In the case of ACP coding *cbxD*, it can be only substituted by *natAC1* but not by *natAC2*; and for the AMP‐dependent synthetase‐ligase coding *cbxC,* the correct substitute is *natAL1* but not *natAL2*. The exception to this general rule is the aminohydrolase coding *cbxE* that cannot be backed up by *natAM*. This result indicates the essential role of this activity in the biosynthesis of benzoxazoles. Amidohydrolases CbxE and NatAM might participate by themselves or in combination with other enzymatic activities in the choice of precursors to be incorporated into the final benzoxazole moiety during the biosynthesis of caboxamycin and nataxazole.

## Conclusions

*Streptomyces* sp. NTK937 presents a versatile metabolism for the biosynthesis of benzoxazole compounds of the caboxamycin type. This includes the presence of several paralogues of the caboxamycin biosynthesis structural genes and others that can interact by cross‐talk with the caboxamycin pathway. This metabolic network allows the production of at least two caboxamycin derivatives: *O*‐methylcaboxamycin and 3′‐hydroxycaboxamycin. The metabolic versatility of this pathway might allow the generation of novel caboxamycin derivatives using different biotechnological approaches involving genetic engineering such as combinatorial biosynthesis and mutasynthesis.

## Experimental procedures

### Strains, culture conditions and plasmids

*Streptomyces* sp. NTK937 (Hohmann *et al*., 2009), producer of caboxamycin; *Streptomyces* sp. Tü6176 (Sommer *et al*., 2008), producer of nataxazole; *Streptomyces albus* J1074 (Chater and Wilde, 1976) and *Streptomyces lividans* JT46 (Tsai and Chen, 1987) were used as heterologous hosts for gene and cluster expression, respectively; *E. coli* DH10B (Invitrogen, Pfullingen, Germany) and *E. coli* ET12567 (pUB307) (Kieser *et al*., 2000) were used for subcloning and intergeneric conjugation respectively. Yeast strain *Saccharomyces cerevisiae* VL6‐48 (*MAT α, his3‐Δ200, trp1‐D1, ura3‐52, lys2, ade2‐101, met14, psi+ cir* ^*0*^) was used for transformation‐associated recombination (TAR) cloning. For *Streptomyces* species, tryptone soy broth (TSB) was used for growth, MA for conjugation and sporulation and R5A for secondary metabolite production (Fernández *et al*., 1998). *E. coli* culture media LB and 2xTY were used as described (Green and Sambrook, 2012). Yeast transformants were selected on YNB‐trp medium, and yeast colonies were cultured overnight in YPD medium (Cano‐Prieto *et al*., 2015a).

Where plasmid‐bearing or mutant strains were used, media were supplemented with their due antibiotic: ampicillin (100 μg ml^−1^), apramycin (100 μg ml^−1^ for *E. coli*, 25 μg ml^−1^ for *Streptomyces*), thiostrepton (50 μg ml^−1^), hygromycin (50 μg ml^−1^), kanamycin (25 μg ml^−1^), tetracycline (10 μg ml^−1^), chloramphenicol (25 μg ml^−1^) and/or nalidixic acid (50 μg ml^−1^).

Two main plasmids were used: pEM4T (Menéndez *et al*., 2006) for gene expression and pEFBAoriT (Horna *et al*., 2011) for gene replacement. pCR‐Blunt (Invitrogen) was used for routine PCR product cloning for verification. Yeast/*E. coli* shuttle‐actinobacterial chromosome integrative vector pCAP01 (Yamanaka *et al*., 2014) was used both for the TAR‐cloning process and as a source of the kanamycin resistance *aph(3)II* gene. pSETeTc (Cano‐Prieto *et al*., 2015a) was used as source of the thiostrepton resistance *tsr* gene, and pLHyg (Olano *et al*., 2004) as the source of the hygromycin resistance *hyg* gene.

### DNA manipulation

Database searching and sequence analysis were carried out with the bioinformatics tools antiSMASH (Blin *et al*., 2013; Weber *et al*., 2015) and BLAST (Altschul *et al*., 1997). DNA manipulations were performed according to standard procedures for *E. coli* (Green and Sambrook, 2012) and *Streptomyces* (Kieser *et al*., 2000). PCR amplifications were carried out with Herculase II Fusion DNA Polymerase (Agilent Technologies, Madrid, Spain) following an optimized standard PCR procedure on a SureCycler 8800 thermocycler (Agilent Technologies): initial denaturation at 99.9°C for 2 min, 30 cycles comprised of 99.9°C denaturation for 10 s, 65°C annealing for 20 s and 72°C elongation at 30 s per kb of DNA to be amplified, plus an extra final cycle of 72°C for 3 min. Products of the expected size were cloned into pCR‐Blunt for sequence verification. All oligonucleotides used in this work are shown in Tables S1–S3 (Supporting information). PCR products were subsequently cloned into appropriate vectors using the selected restriction sites incorporated in the oligonucleotides.

### Construction of plasmids

Gene replacement pEFBAoriT plasmids were constructed by ligating the corresponding ~2 kb up‐ and downstream fragments flanking the apramycin resistance gene *aac(3)IV* in the plasmid, followed by cloning of the *tsr* gene, taking advantage of a XbaI restriction site located immediately before *oriT*. These constructions were introduced into *Streptomyces* sp. NTK937 through intergeneric conjugation from *E. coli* ET12567(pUB307) and then screened simultaneously for apramycin resistance and thiostrepton sensitivity, indication of a successful double cross‐over event. In pEFBAoriT plasmids for the creation of double mutant strains, an intermediate step is required, where *aac(3)IV* is excised and replaced by *tsr* using the NheI additional sites included in the corresponding oligonucleotides, followed by introduction of *hyg* as a SpeI‐NheI fragment in the same XbaI site as previously described. Double mutants were sought in the same fashion as single ones and likewise screened for thiostrepton resistance and hygromycin sensitivity. The plasmids for triple mutant generation were built similarly, excising *aac(3)IV* in favour of *hyg*, then using *aph(3)II* as the second marker and screening colonies for hygromycin resistance and kanamycin sensitivity. All mutants were further confirmed by PCR amplification of the replaced gene, given the differing sizes of the antibiotic resistance gene versus the original *orf*. In cases where the difference in size was < 100 bp (*cbxC, cbxE, cbxF’*), additional differential enzymatic digestion was performed (Supporting information, Fig. S13).

Gene expression plasmids derived of pEM4T were constructed by appropriate digestion and directed ligation using the available BamHI and EcoRI sites. Where cohesive ends were not available, fragments and vector were previously made blunt‐ended according to the protocol for Platinum^®^ Pfx DNA polymerase (Thermo Fisher Scientific, Barcelona, Spain). After intergeneric conjugation, colonies were screened for thiostrepton resistance. Overexpression plasmids derived of pEM4HT (pHT‐) and pEM4KT (pKT‐), used for complementation assays (Supporting information, Table S2) in double and triple mutant strains, were created by *a posteriori* opening of the final construction at the sole EcoRV site in the middle of the *tsr* gene, and blunt‐ended ligation of the *hyg* or *aph(3)II* gene, respectively, and then introduced as well by conjugation. Overexpression plasmids containing genes from the nataxazole biosynthesis cluster were readily used when available (Cano‐Prieto *et al*., 2015a), or generated as described here for the genes not available (Supporting information, Table S2).

Heterologous expression of the entire cluster was generated on the trifunctional pCAP01 vector according to protocol (Yamanaka *et al*., 2014). Oligonucleotide pairs CABTAR/AB and CABTAR/CD were used to generate the capturing arms and then ligated into pCAP01 to generate capture plasmid pCAB. BamHI‐linearized pCAB and MfeI‐PstI‐digested (restriction sites loosely flanking the caboxamycin cluster region) *Streptomyces* sp. NTK937 genomic DNA were co‐transformed into *Saccharomyces cerevisiae* VL6‐48 following the LiAc/PEG/ssDNA method (Gietz and Schiestl, 2007). Yeast transformants were selected on YNB‐trp medium and screened by PCR for the presence of caboxamycin biosynthetic genes. Positive colonies were grown on YPD medium overnight and subjected to glass beads DNA extraction (Hoffman and Winston, 1987). The resulting construction, pCABTAR, was then introduced into *Streptomyces lividans* JT46 by protoplast transformation (Kieser *et al*., 2000).

### Analysis of metabolites by UPLC and HPLC‐MS

Cultures of selected strains or mutants were extracted with ethyl acetate containing 1% formic acid (to enhance the extraction of compounds containing ionizing groups) and analysed by reverse phase chromatography with an Acquity UPLC instrument fitted with a BEH C18 column (1.7 μm, 2.1 × 100 mm; Waters, Barcelona, Spain), using acetonitrile (AcN) and aqueous 0.1% trifluoroacetic acid (TFA) as eluents. The program uses an isocratic hold of 10% AcN for 1 min, followed by a linear gradient up to 100% AcN over 7 min, at a flow rate of 0.5 ml min^−1^ and a column temperature of 35°C.

For HPLC‐MS analysis, an Alliance chromatographic system coupled to a ZQ4000 mass spectrometer and a SunFire C18 column (3.5 μm, 2.1 × 150 mm; Waters) was used. Solvents were the same as above, and elution was performed with an initial isocratic hold with 10% AcN during 4 min followed by a linear gradient of AcN (10% to 88%) over 30 min, all at 0.25 ml min^−1^. MS analysis was carried out by positive mode electrospray ionization (ESI), with a capillary voltage of 3 kV and a cone voltage of 20 V. Spectral identification and characterization of peaks were performed in both cases by photodiode array detection at 330 nm, using Empower software (Waters) to extract bidimensional chromatograms at different wavelengths, depending on the spectral characteristics of the desired compound.

### Isolation and structural characterization of compounds

Liquid production cultures were generally incubated at 30°C and 250 rpm for 7 days, and then, 1 ml of samples from each of the flasks was extracted with an equal volume of acidified ethyl acetate. Solid production cultures were carried out using 25‐well plates with 1.5 ml solid R5A medium each and inoculated with a sterile cotton swab, then incubated at 30°C for 7 days and extracted with an equal volume of acidified ethyl acetate. Both types of samples were subsequently vacuum‐dried and redissolved in 50:50 DMSO:MeOH before chromatographic analysis.

Products **1** – **3** were isolated from 5 × 400 ml (in 2 l flasks) cultures whose supernatants were first filtered, then concentrated on a C18 cartridge (10 g; Waters) and subsequently fractioned on a 0.1%TFA–MeOH gradient. Fractions were submitted to UPLC analysis, and the fractions containing desired compounds were dried *in vacuo*, resuspended in 50:50 DMSO:MeOH and processed on a preparative HPLC SunFire C18 column (10 μm, 10 × 250 mm; Waters) using experimentally determined isocratic mixtures of 0.05%TFA with either AcN or MeOH at 5 ml min^−1^. The purity of the isolated peaks was determined by HPLC‐MS before structural elucidation. The isolated compounds were then dried *in vacuo*, resuspended in 50:50 *tert*‐butanol:water and lyophilized. Structural elucidation was carried out by a combination of ^1^H, ^13^C, COSY and HSQC experiments using DMSO‐*d*_*6*_ as solvent (Supporting information, Fig. S14 to S16 and Tables S4 and S5).

### Bioactivity assays

The antibiotic activities of **1** – **3** were analysed with an antibiotic disc diffusion assay against *S. albus* J1074, *E. coli*,* Staphylococcus aureus* and *Micrococcus luteus*. The antifungal activity was tested against *Candida albicans*. In all cases, 1, 2.5, 5, 10 and 20 μg of each compound was used. Plates were incubated overnight at 37°C for *E. coli*,* S. aureus* and *M. luteus* and at 30°C for *S. albus* J1074 and *C. albicans*.

Cytotoxic activity of compounds **1** – **3** was tested against the following human tumour cell lines: colon adenocarcinoma (HT29), non‐small cell lung cancer (A549), breast adenocarcinoma (MDA‐MB‐231), gastric carcinoma (AGS) and ovarian carcinoma (A2780). Mouse embryonic fibroblast cell line NIH/3T3 was used as control to evaluate cytotoxicity against non‐malignant cells. Cells were previously grown for a week on DMEM‐10% FBS medium, then aliquoted to 5000 cells per well in 96‐well plates using the *Cell counting kit‐8‐(96992)* (Sigma‐Aldrich, Barcelona, Spain) and grown for an extra 24 h. Compounds were dissolved in DMSO, keeping in mind that final concentration of DMSO in the assays should be kept at 0.1%. After the incubation, 10 μl of compound (in diverse concentrations) was added to each well and incubated for another 48 h. Lastly, 10 μl of CCK‐8 reagent (Sigma‐Aldrich) was added, left to develop for 2 h in the incubator and measured at 450 nm using an *Elisa Bio‐tek ELx 800* (BioTek, Winooski, VT, United States).

## Conflict of interest

None declared.

## Supporting information


**Fig. S1.** UPLC analysis showing production of salicylic acid (**4**) in cultures of *S. albus* J1074 carrying pEM4T, pT‐SS (*cbxA*), pT‐SSALT (*cbxA’*) or pT‐SSTu (CF54_20720).
**Fig. S2.** UPLC analysis of *Streptomyces* sp. NTK937 wild‐type (WT) and mutant strains.
**Fig. S3.** (A) Genetic complementation of ΔcbxR mutant strain.
**Fig. S4.** Genetic complementation of ΔcbxA mutant strain.
**Fig. S5.** Genetic complementation of ΔcbxB mutant strain.
**Fig. S6.** Genetic complementation of ΔcbxC/C’ mutant strain.
**Fig. S7.** Genetic complementation of ΔcbxD mutant strain.
**Fig. S8.** Genetic complementation of ΔcbxE mutant strain.
**Fig. S9.** Genetic complementation of ΔcbxG/[FGHI]’/entA mutant strain.
**Fig. S10.** Genetic complementation of ΔcbxH/[FGHI]’/entB mutant strain.
**Fig. S11.** Genetic complementation of ΔcbxI/[FGHI]’ mutant strain.
**Fig. S12.** (A) UPLC analysis of Streptomyces sp. NTK937 wild‐type strain (WT) and DAHP synthase‐deficient mutant strains. B) UPLC analysis of *Streptomyces* sp. NTK937 wild‐type strain (WT) and DAHP synthase‐deficient mutant strains in cultures supplemented with each individual aromatic aminoacid or a mixture of all three.
**Fig. S13.** PCR analysis of *Streptomyces* sp. NTK 937 mutant strains.
**Fig. S14.** ^1^H NMR spectrum of *O*‐methylcaboxamycin (**2**).
**Fig. S15**. ^1^H NMR spectrum of 3′‐hydroxycaboxamycin (**3**).
**Fig. S16**. Expansion of the aromatic region in the proton spectrum of 3′‐hydroxycaboxamycin (**3**).
**Table S1.** Primers used in this work for the amplification of DNA regions used in gene inactivation experiments.
**Table S2.** Primers used in this work for the amplification of DNA regions used in gene expression experiments.
**Table S3.** Primers used in this work for the amplification of resistance genes and heterologous expression of caboxamycin biosynthesis gene cluster.
**Table S4.** *O*‐methylcaboxamycin (**2**) ^13^C and ^1^H NMR data acquired in DMSO‐*d*_*6*._
**Table S5.** 3′‐hydroxycaboxamycin (**3**) ^13^C and ^1^H NMR data acquired in DMSO‐*d*_*6*_ (500 MHz, 24°C).

## References

[mbt212716-bib-0001] Altschul, S.F. , Madden, T.L. , Schäffer, A.A. , Zhang, J. , Zhang, Z. , Miller, W. , and Lipman, D.J. (1997) Gapped BLAST and PSI‐BLAST: a new generation of protein database search programs. Nucl Acids Res 25: 3389–3402. 9254694 10.1093/nar/25.17.3389 PMC146917

[mbt212716-bib-0002] Blin, K. , Medema, M.H. , Kazempour, D. , Fischbach, M.A. , Breitling, R. , Takano, E. , and Weber, T. (2013) AntiSMASH 2.0 – a versatile platform for genome mining of secondary metabolite producers. Nucl Acids Res 41: W204–W212. 23737449 10.1093/nar/gkt449 PMC3692088

[mbt212716-bib-0003] Cano‐Prieto, C. , García‐Salcedo, R. , Sánchez‐Hidalgo, M. , Braña, A.F. , Fiedler, H.P. , Méndez, C. , *et al* (2015a) Genome mining of *Streptomyces* sp. Tü6176: characterization of the nataxazole biosynthesis pathway. ChemBioChem 16: 1461–1473. 25892546 10.1002/cbic.201500153

[mbt212716-bib-0004] Cano‐Prieto, C. , Losada, A.A. , Braña, A.F. , Méndez, C. , Salas, J.A. , and Olano, C. (2015b) Crosstalk of nataxazole pathway with chorismate‐derived ionophore biosynthesis pathways in *Streptomyces* sp. Tü6176. ChemBioChem 16: 1925–1932. 10.1002/cbic.201500261 26083234

[mbt212716-bib-0005] Chater, K.F. , and Wilde, L.C. (1976) Restriction of a bacteriophage of *Streptomyces albus* G involving endonuclease SalI. J Bacteriol 128: 644–650. 977549 10.1128/jb.128.2.644-650.1976 PMC232802

[mbt212716-bib-0006] Culbertson, J.E. , and Toney, M.D. (2013) Expression and characterization of PhzE from *P. aeruginosa* PAO1: aminodeoxyisochorismate synthase involved in pyocyanin and phenazine‐1‐carboxylate production. Biochim Biophys Acta 1834: 250–256. 10.1016/j.bbapap.2012.10.010 23099261

[mbt212716-bib-0007] De Crécy‐Lagard, V. , Blanc, V. , Gil, P. , Naudin, L. , Lorenzon, S. , Famechon, A. , *et al* (1997) Pristinamycin I biosynthesis in *Streptomyces pristinaespiralis*: molecular characterization of the first two structural peptide synthase genes. J Bacteriol 179: 705–713. 9006024 10.1128/jb.179.3.705-713.1997 PMC178751

[mbt212716-bib-0008] Engelhardt, K. , Degnes, K.F. , and Zotchev, S.B. (2010) Isolation and characterization of the gene cluster for biosynthesis of the thiopeptide antibiotic TP‐1161. Appl Environ Microbiol 76: 7093–7101. 20851988 10.1128/AEM.01442-10 PMC2976260

[mbt212716-bib-0009] Fernández, E. , Weissbach, U. , Reillo, C.S. , Braña, A.F. , Méndez, C. , Rohr, J. , and Salas, J.A. (1998) Identification of two genes from *Streptomyces argillaceus* encoding glycosyltransferases involved in transfer of a disaccharide during biosynthesis of the antitumor drug mithramycin. J Bacteriol 180: 4929–4937. 9733697 10.1128/jb.180.18.4929-4937.1998 PMC107519

[mbt212716-bib-0010] Gaitatzis, N. , Kunze, B. , and Müller, R. (2001) *In vitro* reconstitution of the myxochelin biosynthetic machinery of *Stigmatiella aurantica* Sg a15: biochemical characterization of a reductive release mechanism for nonribosomal peptide synthetases. Proc Nat Ac Sci USA 98: 11136–11141. 10.1073/pnas.201167098 PMC58696 11562468

[mbt212716-bib-0011] Gietz, R.D. , and Schiestl, R.H. (2007) High‐efficiency yeast transformation using the LiAc/SS carrier DNA/PEG method. Nat Protoc 2: 31–34. 17401334 10.1038/nprot.2007.13

[mbt212716-bib-0012] Green, M.R. , and Sambrook, J. (2012) Molecular Cloning: A Laboratory Manual. New York, USA: Cold Spring Harbor Laboratory Press.

[mbt212716-bib-0013] Gross, H. , and Loper, J.E. (2009) Genomics of secondary metabolite production by *Pseudomonas* spp. Nat Prod Rep 26: 1408–1446. 19844639 10.1039/b817075b

[mbt212716-bib-0014] Hoffman, C.S. , and Winston, F. (1987) A ten‐minute DNA preparation from yeast efficiently releases autonomous plasmids for transformation of *Escherichia coli* . Gene 57: 267–272. 3319781 10.1016/0378-1119(87)90131-4

[mbt212716-bib-0015] Hohmann, C. , Schneider, K. , Bruntner, C. , Irran, E. , Nicholson, G. , Bull, A.T. , *et al* (2009) Caboxamycin, a new antibiotic of the benzoxazole family produced by the deep‐sea strain *Streptomyces* sp.NTK 937. J Antibiot 62: 99–104. 19198633 10.1038/ja.2008.24

[mbt212716-bib-0016] Horna, D.H. , Gómez, C. , Olano, C. , Palomino‐Schätzlein, M. , Pineda‐Lucena, A. , Carbajo, R.J. , *et al* (2011) Biosynthesis of the RNA polymerase inhibitor streptolydigin in *Streptomyces lydicus*: tailoring modification of 3‐methylaspartate. J Bacteriol 193: 2647–2651. 21398531 10.1128/JB.00108-11 PMC3133142

[mbt212716-bib-0017] Huang, S. , Tong, M.H. , Qin, Z. , Deng, Z. , Deng, H. , and Yu, Y. (2015) Identification and characterization of the biosynthetic gene cluster of thiolutin, a tumor angiogenesis inhibitor, in *Saccharothrix algeriensis* NRRL B‐24137. Anticancer Agents Med Chem 15: 277–284. 25353334 10.2174/1871520614666141027145200

[mbt212716-bib-0018] Janata, J. , Kadlcik, S. , Koberska, M. , Ulanova, D. , Kamenik, Z. , Novak, P. , *et al* (2015) Lincosamide synthetase – a unique condensation system combining elements of nonribosomal peptide synthetase and mycothiol metabolism. PLoS ONE 10: e0118850. 25741696 10.1371/journal.pone.0118850 PMC4351081

[mbt212716-bib-0019] Karki, S. , Kwon, S.Y. , Yoo, H.G. , Suh, J.W. , Park, S.H. , and Kwon, H.J. (2010) The methoxymalonyl‐acyl carrier protein biosynthesis locus and the nearby gene with the β‐ketoacyl synthase domain are involved in the biosynthesis of galbonolides in *Streptomyces galbus*, but these loci are separate from the modular polyketide synthase gene cluster. FEMS Microbiol Lett 310: 69–75. 20662933 10.1111/j.1574-6968.2010.02048.x

[mbt212716-bib-0020] Kerbarh, O. , Ciulli, A. , Howard, N.I. , and Abell, C. (2005) Salicylate biosynthesis: overexpression, purification and characterization of Irp9, a bifunctional salicylate synthase from *Yersinia enterocolitica* . J Bacteriol 187: 5061–5066. 16030197 10.1128/JB.187.15.5061-5066.2005 PMC1196042

[mbt212716-bib-0021] Kieser, T. , Bibb, M.J. , Buttner, M.J. , Chater, K.F. and Hopwood, D.A. (2000) Practical Streptomyces Genetics. Norwich, UK: The John Innes Foundation.

[mbt212716-bib-0022] Li, W. , Khullar, A. , Chou, S.C. , Sacramo, A. , and Gerratana, B. (2009) Biosynthesis of sibiromycin, a potent antitumor antibiotic. Appl Environ Microbiol 75: 2869–2878. 19270142 10.1128/AEM.02326-08 PMC2681668

[mbt212716-bib-0023] Light, S.H. , and Anderson, W.F. (2013) The diversity of allosteric controls at the gateway to aromatic amino acid biosynthesis. Protein Sci 22: 395–404. 23400945 10.1002/pro.2233 PMC3610045

[mbt212716-bib-0024] Lv, M. , Zhao, J. , Deng, Z. , and Yu, Y. (2015) Characterization of the biosynthetic gene cluster for benzoxazole antibiotics A33853 reveals unusual assembly logic. Chem Biol 22: 1313–1324. 26496684 10.1016/j.chembiol.2015.09.005

[mbt212716-bib-0025] Menéndez, N. , Nur‐e‐Alam, M. , Fischer, C. , Braña, A.F. , Salas, J.A. , Rohr, J. , and Méndez, C. (2006) Deoxysugar transfer during chromomycin A3 biosynthesis in *Streptomyces griseus* subsp. *griseus*: new derivatives with antitumor activity. Appl Environ Microbiol 72: 167–177. 16391039 10.1128/AEM.72.1.167-177.2006 PMC1352227

[mbt212716-bib-0026] Michel, K.H. , Boeck, L.D. , Hoehn, M.M. , Jones, N.D. , and Chaney, M.O. (1984) The discovery, fermentation, isolation, and structure of antibiotic A33853 and its tetraacetyl derivative. J Antibiot 37: 441–445. 6547431 10.7164/antibiotics.37.441

[mbt212716-bib-0027] Olano, C. , Wilkinson, B. , Sánchez, C. , Moss, S.J. , Sheridan, R. , Math, V. , *et al* (2004) Biosynthesis of the angiogenesis inhibitor borrelidin by *Streptomyces parvulus* Tü4055: cluster analysis and assignment of functions. Chem Biol 11: 87–97. 15112998 10.1016/j.chembiol.2003.12.018

[mbt212716-bib-0028] Olano, C. , Cano‐Prieto, C. , Losada, A.A. , Bull, A.T. , Goodfellow, M. , Fiedler, H.P. , *et al* (2014) Draft genome sequence of marine actinomycete *Streptomyces* sp. strain NTK 937, producer of the benzoxazole antibiotic caboxamycin. Genome Announc 2: e00534–14. 24994793 10.1128/genomeA.00534-14 PMC4081993

[mbt212716-bib-0029] Pickens, L.B. , Kim, W. , Wang, P. , Zhou, H. , Watanabe, K. , Gomi, S. , and Tang, Y. (2010) Biochemical analysis of the biosynthetic pathway of an anticancer tetracycline SF2575. J Am Chem Soc 131: 17677–17689. 10.1021/ja907852c PMC2800175 19908837

[mbt212716-bib-0030] Praseuth, A.P. , Wang, C.C.C. , Watanabe, K. , Hotta, K. , Oguri, H. , and Oikawa, H. (2008) Complete sequence of biosynthetic gene cluster responsible for producing triostin A and evaluation of quinomycin‐type antibiotics from *Streptomyces triostinicus* . Biotechnol Prog 24: 1226–1231. 19194935 10.1002/btpr.34

[mbt212716-bib-0031] Rui, Z. , Ye, M. , Wang, S. , Fujikawa, K. , Akerele, B. , Aung, M. , *et al* (2012) Insights into a divergent phenazine biosynthetic pathway governed by a plasmid‐borne esmeraldin gene cluster. Chem Biol 19: 1116–1125. 22999880 10.1016/j.chembiol.2012.07.025

[mbt212716-bib-0032] Sato, S. , Kajiura, T. , Noguchi, M. , Takehana, K. , Kobayashi, T. , and Tsuji, T. (2001) AJI9561, a new cytotoxic benzoxazole derivative produced by *Streptomyces* sp. J Antibiot 54: 102–104. 11269707 10.7164/antibiotics.54.102

[mbt212716-bib-0033] Singh, S. , Veeraswamy, G. , Bhattarai, D. , Goo, J.I. , Lee, K. , and Choi, Y. (2015) Recent advances in the development of pharmacologically active compounds that contain a benzoxazole scaffold. Asian J Org Chem 4: 1338–1361.

[mbt212716-bib-0034] Sommer, P.S. , Almeida, R.C. , Schneider, K. , Beil, W. , Süssmuth, R.D. , and Fiedler, H.P. (2008) Nataxazole, a new benzoxazole derivative with antitumor activity produced by *Streptomyces* sp. Tü 6176. J Antibiot 61: 683–686. 19168984 10.1038/ja.2008.97

[mbt212716-bib-0035] Tahlan, K. , Park, H.U. , Wong, A. , Beatty, P.H. , and Jensen, S.E. (2004) Two sets of paralogous genes encode the enzymes involved in the early stages of clavulanic acid and clavam biosynthesis in *Streptomyces clavuligerus* . Antimicrob Agents Chemother 48: 930–939. 14982786 10.1128/AAC.48.3.930-939.2004 PMC353097

[mbt212716-bib-0036] Talley, D.C. , Delang, L. , Neyts, J. , Leyssen, P. , and Smith, P.J. (2016) Exploring the importance of zinc binding and steric/hydrophobic factors in novel HCV replication inhibitors. Bioorg Med Chem Lett 26: 1196–1199. 26804234 10.1016/j.bmcl.2016.01.035

[mbt212716-bib-0037] Tsai, J.F.Y. , and Chen, C.W. (1987) Isolation and characterization of *Streptomyces lividans* mutants deficient in intraplasmid recombination. Mol Gen Genet 208: 211–218. 3112520 10.1007/BF00330444

[mbt212716-bib-0038] Ueki, M. , Ueno, K. , Miyadoh, S. , Abe, K. , Shibata, K. , Taniguchi, M. , and Oi, S. (1993) UK‐1, a novel cytotoxic metabolite from *Streptomyces* sp. 517‐02. I. Taxonomy, fermentation, isolation, physico‐chemical and biological properties. J Antibiot 46: 1089–1094. 8360104 10.7164/antibiotics.46.1089

[mbt212716-bib-0039] Weber, T. , Blin, K. , Duddela, S. , Krug, D. , Kim, H.U. , Bruccoleri, R. , *et al* (2015) AntiSMASH 3.0‐a comprehensive resource for the genome mining of biosynthetic gene clusters. Nucleic Acids Res 43: W237–W243. 25948579 10.1093/nar/gkv437 PMC4489286

[mbt212716-bib-0040] Wu, Q. , Liang, J. , Lin, S. , Zhou, X. , Bai, L. , Deng, Z. , and Wang, Z. (2011) Characterization of the biosynthesis gene cluster for the pyrrole polyether antibiotic calcimycin (A23187) in *Streptomyces chartreusis* NRRL 3882. Antimicrob Agents Chemother 55: 974–982. 21173184 10.1128/AAC.01130-10 PMC3067094

[mbt212716-bib-0041] Yamanaka, K. , Reynolds, K.A. , Kersten, R.D. , Ryan, K.S. , Gonzalez, D.J. , Nizet, V. , *et al* (2014) Direct cloning and refactoring of a silent lipopeptide biosynthetic gene cluster yields the antibiotic taromycin A. Proc Natl Acad Sci USA 111: 1957–1962. 24449899 10.1073/pnas.1319584111 PMC3918841

