# Supplementary material for: Caboxamycin biosynthesis pathway and identification of novel benzoxazoles produced by cross‐talk in Streptomyces sp. NTK 937
Source: Microb Biotechnol. 2017 Apr 18;10(4):873–85. doi: 10.1111/1751-7915.12716 (PMC5481532; doi:10.1111/1751-7915.12716)
Supplement: Supplementary file 1 — Fig. S1. UPLC analysis showing production of salicylic acid (4) in cultures of S. albus J1074 carrying pEM4T, pT‐SS (cbxA), pT‐SSALT (cbxA’) or pT‐SSTu (CF54_20720). Fig. S2. UPLC analysis of Streptomyces sp. NTK937 wild‐type (WT) and mutant strains. Fig. S3. (A) Genetic complementation of ΔcbxR mutant strain. Fig. S4. Genetic complementation of ΔcbxA mutant strain. Fig. S5. Genetic complementation of ΔcbxB mutant strain. Fig. S6. Genetic complementation of ΔcbxC/C’ mutant strain. Fig. S7. Genetic complementation of ΔcbxD mutant strain. Fig. S8. Genetic complementation of ΔcbxE mutant strain. Fig. S9. Genetic complementation of ΔcbxG/[FGHI]’/entA mutant strain. Fig. S10. Genetic complementation of ΔcbxH/[FGHI]’/entB mutant strain. Fig. S11. Genetic complementation of ΔcbxI/[FGHI]’ mutant strain. Fig. S12. (A) UPLC analysis of Streptomyces sp. NTK937 wild‐type strain (WT) and DAHP synthase‐deficient mutant strains. B) UPLC analysis of Streptomyces sp. NTK937 wild‐type strain (WT) and DAHP synthase‐deficient mutant strains in cultures supplemented with each individual aromatic aminoacid or a mixture of all three. Fig. S13. PCR analysis of Streptomyces sp. NTK 937 mutant strains. Fig. S14. 1H NMR spectrum of O‐methylcaboxamycin (2). Fig. S15. 1H NMR spectrum of 3′‐hydroxycaboxamycin (3). Fig. S16. Expansion of the aromatic region in the proton spectrum of 3′‐hydroxycaboxamycin (3). Table S1. Primers used in this work for the amplification of DNA regions used in gene inactivation experiments. Table S2. Primers used in this work for the amplification of DNA regions used in gene expression experiments. Table S3. Primers used in this work for the amplification of resistance genes and heterologous expression of caboxamycin biosynthesis gene cluster. Table S4. O‐methylcaboxamycin (2) 13C and 1H NMR data acquired in DMSO‐d 6. Table S5. 3′‐hydroxycaboxamycin (3) 13C and 1H NMR data acquired in DMSO‐d 6 (500 MHz, 24°C). [file MBT2-10-873-s001.doc]

**Supplementary material**

**Table S1.** Primers used in this work for the amplification of DNA regions used in gene inactivation experiments.

| **Construct** | **Primer** | **Description^[a]^** | **Nucleotide sequence (5’ 🡪 3’)** | **Mutant strain** |
| --- | --- | --- | --- | --- |
| pSARP-ABt | RSARP/A | EcoRV | TT**GATATC**GCACCCGTGCTCCAGTCG | ΔcbxR |
|  | RSARP/B | BglII | TT**AGATCT**GCCGTGGTCCGACGCC |  |
|  | RSARP/C | NsiI | TTT**ATGCAT**CTCGTGCCGCAGGGTGAG |  |
|  | RSARP/D | SpeI | A**ACTAGT**GCCATGAACTCCTCGACGCC |  |
| pSS-ABt | RSS/A | SpeI | A**ACTAGT**CGGTGAAGTCGGCGACGTG | ΔcbxA |
|  | RSS/B | NsiI | TTT**ATGCAT**GAGGGCAGCCTCAGCCTC |  |
|  | RSS/C | BamHI | AA**GGATCC**GGAACTGGAGGAGACCTGCGA |  |
|  | RSS/D | EcoRV | A**GATATC**TCGAAGCACTCGTCGGCG |  |
| pOA-ABt | ROA/A | SpeI | A**ACTAGT**CGCAGATGCCCACAGGGATCGA | ΔcbxB |
|  | ROA/B | NsiI | AAA**ATGCAT**CGTGGTGTACGGCTCCGGCAG |  |
|  | ROA/C | BglII | AA**AGATCT**GCGCCGTCGTCGAGATCACC |  |
|  | ROA/D | EcoRV | A**GATATC**GCATCATCTCCAGCGAACCGAGTC |  |
| pASL-ABt | RASL/A | SpeI | A**ACTAGT**AGCACACCGAGCGGCACTC | ΔcbxC |
|  | RASL/B | NsiI | TTT**ATGCAT**GTGGTGGACAGCAGGTCCG |  |
|  | RASL/C | BglII | AA**AGATCT**CGGCAAGGTCGACAAGAAGACC |  |
|  | RASL/D | EcoRV | A**GATATC**GTCAGGTCCTTCTCGGCGAC |  |
| pACP-ABt | RACP/A | SpeI | A**ACTAGT**CATGGGCAACTTCGGTGACACGG | ΔcbxD |
|  | RACP/B | NsiI | AAA**ATGCAT**GGTGTGCATCGTGGTCCTGTGTC |  |
|  | RACP/C | BglII | AA**AGATCT**CGGCACCTCGACGCCGTC |  |
|  | RACP/D | EcoRV | A**GATATC**ACGGGAATGAAGGAGCGTGCG |  |
| pAH-ABt | RAH/A | SpeI | A**ACTAGT**CCGTCTCACCTTCGCCGAG | ΔcbxE |
|  | RAH/B | NsiI | ATA**ATGCAT**GTCCACGACGAGCACGGAC |  |
|  | RAH/C | BglII | AA**AGATCT**CGTCGCCGAGAAGGACCTGAC |  |
|  | RAH/D | EcoRV | A**GATATC**GACCACGTTGCAGCGGATG |  |
| pDAHP-ABt | RDAHP/A | SpeI | A**ACTAGT**CACAGCACAGGACAGCGGAG | ΔcbxF |
|  | RDAHP/B | NsiI | TTT**ATGCAT**CGGCGTAACTCGGCCAG |  |
|  | RDAHP/C | BglII | AA**AGATCT**CGCCTACACGACGCTCTGC |  |
|  | RDAHP/D | EcoRV | A**GATATC**GAGGTCGAAGCCGTGCTC |  |
| p23DH-ABt | R23DH/A | SpeI | A**ACTAGT**CGTCCACATCAGCCACTCGC | ΔcbxG |
|  | R23DH/B | NsiI | ATA**ATGCAT**CCAGCAGGGCGACCGAC |  |
|  | R23DH/C | BglII | AA**AGATCT**GGTGCTGTTCCTGCTGTCCG |  |
|  | R23DH/D | EcoRV | A**GATATC**CGCCAGCCAGAAGTCGG |  |
| pIC-ABt | RIC/A | SpeI | A**ACTAGT**CGCTGCCAGGTGGTCCAGG | ΔcbxH |
|  | RIC/B | NsiI | ATA**ATGCAT**CCGAGCAGTGCGTCCACGG |  |
|  | RIC/C | BglII | AA**AGATCT**CGCATGACCCTGGAGTACGTCG |  |
|  | RIC/D | EcoRV | A**GATATC**GGAGGTCACGCAGGAGGGC |  |
| pAS-ABt | RAS/A | SpeI | A**ACTAGT**CGGCAGGCTCACGCTGATC | ΔcbxI |
|  | RAS/B | NsiI | ATT**ATGCAT**CCATCAGTTCGAGCCGGTCGG |  |
|  | RAS/C | BglII | TT**AGATCT**GCGTGACCTCCTGCTCGG |  |
|  | RAS/D | EcoRV | A**GATATC**CGCTACCGTTGCTCGTACCG |  |
| pSSALT-AtBh | RSSALT/A | SpeI | A**ACTAGT**CCTCCTGATCGTCCTCGTCCTG | ΔcbxA’ |
|  | RSSALT/B | NsiI/NheI | TTT**ATGCATGCTAGC**CCTCCGTCTCGTAGACCATG |  |
|  | RSSALT/C | BglII/NheI | AA**AGATCTGCTAGC**GAGGAGACCTGCGAGAAGCTGC |  |
|  | RSSALT/D | EcoRV | A**GATATC**GTCGAGGAGTCCACGGGACG |  |
| pASLALT-AtBh | RASLALT/A | SpeI | A**ACTAGT**CTGGTCGAACTACCACGAGGTCG | ΔcbxC’ |
|  | RASLALT/B | NsiI/NheI | TTT**ATGCATGCTAGC**TGGGCACGGTAGGTGGC |  |
|  | RASLALT/C | BamHI/NheI | AA**GGATCCGCTAGC**CGCCTACAAACTCCCGGACC |  |
|  | RASLALT/D | EcoRV | T**GATATC**TGCCACCGAGTTCCGTGC |  |
| pDAHP-AS-ALT-AtBh | RASALT/A | SpeI | A**ACTAGT**CCAGGTGCGGACTGCTCAG | Δcbx  [FGHI]’ |
|  | RASALT/B | NsiI/NheI | TTA**ATGCAT**A**GCTAGC**GAATCGGTCCTCACGCGGGAC |  |
|  | RDAHPAL/C | BglII/NheI | AA**AGATCTGCTAGC**CAGTTCCGTTCTGACCCGC |  |
|  | RDAHPAL/D | EcoRVI | A**GATATC**GGTAGTCGTGAGAGGTAGCGTGCC |  |
| pDAHPALT-AtBh | RDAHPALT/A | SpeI | A**ACTAGT**CGCTTGAGGACGAAGTTGGAGCC | ΔcbxF’ |
|  | RDAHPALT/B | NsiI/NheI | TTT**ATGCATGCTAGC**GGCGTACACGACCTTCTGCGAC |  |
|  | RDAHPALT/C | BglII/NheI | AA**AGATCTGCTAGC**CAGTTCCGTTCTGACCCGC |  |
|  | RDAHPALT/D | EcoRV | A**GATATC**GGTAGTCGTGAGAGGTAGCGTGCC |  |
| pDAHPbis-AhBk | RDAHPbis/A | SpeI | A**ACTAGT**CAACGACTCGAAGGACGTGGTGG | ΔcbxF’’ |
|  | RDAHPbis/B | NsiI/NheI | TTT**ATGCATGCTAGC**TGAACCGCAGCCAGTCCCTG |  |
|  | RDAHPbis/C | BglII/NheI | AA**AGATCTGCTAGC**GGATAGGTTTCGAGGTCCG |  |
|  | RDAHPbis/D | EcoRV | T**GATATC**TGTTGTAGAAGCCGACGGTCTC |  |
| pASLm7-AtBh | RASLm7/A | SpeI | A**ACTAGT**GCTTCGTCGAGCCCATCCAG | Δorf-7 |
|  | RASLm7/B | NsiI/NheI | TTT**ATGCATGCTAGC**GTCGTAGCGGAGGGTCTCCTC |  |
|  | RASLm7/C | BglII/NheI | AA**AGATCTGCTAGC**GGAGATGTTCGTCGAAGGATGGTCAC |  |
|  | RASLm7/D | EcoRV | A**GATATC**GAGCAGGCCGTCCACATC |  |
| pUP-ABt | RUP/A | SpeI | A**ACTAGT**ACTCGTGGATCAGCAGGAACAGCAG | Δorf-3-2-1 |
|  | RUP/B | NsiI | TTT**ATGCAT**CCGCACGGCTGGACGTTC |  |
|  | RUP/C | BglII | AA**AGATCT**ACTCAACGTCATGCTCGGCGTG |  |
|  | RUP/D | EcoRV | A**GATATC**AGCAGCTCTACACCTATGTCTCACGG |  |
| pDOWN-ABt | RDOWN/A | SpeI | A**ACTAGT**CGACCGGCTCGAACTGATGGTG | Δorf+1+2+3 |
|  | RDOWN/B | NsiI | TTT**ATGCAT**CCGACCTGACGACGGTGGC |  |
|  | RDOWN/C | BglII | AA**AGATCT**GACAAGGAGTCGTCGGACGAGCAG |  |
|  | RDOWN/D | EcoRV | A**GATATC**ACGTCCTCGCCTTCCAGGAGAAG |  |
| pTet-ABt | RTet/A | SpeI | A**ACTAGT**GGCAACGAGGTACGGATCGTGGAC | Δorf-5 |
|  | RTet/B | NsiI | TTA**ATGCAT**ACGGCACCGCTGAGCGAC |  |
|  | RTet/C | BglII | AA**AGATCT**CCCAGCCGTTCTGGTCGATG |  |
|  | RTet/D | EcoRV | A**GATATC**CTCTTCGGTGACCGGAGGC |  |
| pMer-ABt | RMer/A | SpeI | A**ACTAGT**GGAAGAGCAGATAGTCCTGGAAGACC | Δorf+4 |
|  | RMer/B | NsiI | ATT**ATGCAT**GGAGACCACCGATGAGCGTAC |  |
|  | RMer/C | BglII | AA**AGATCT**GGACTCACGCCGAGCAGCC |  |
|  | RMer/D | EcoRV | A**GATATC**AGGTCACCGAGCTGAAGGAAGCC |  |
| pDHENT-AhBk | RDHENT/A | EcoRV | A**GATATC**CTCGTCCACTCCGCAGATCTC | ΔentA |
|  | RDHENT/B | NdeI/NheI | AATATAA**CATATG**A**GCTAGC**ACATCACCATGCACGACCTGTAC |  |
|  | RDHENT/C | PstI/NheI | ATAT**CTGCAGCTAGC**CGTCACGAGGGCCAGCC |  |
|  | RDHENT/D | SpeI | A**ACTAGT**GACGACGCTGAGCAGTGACATCGC |  |
| pICENT-AhBk | RICENT/A | EcoRV | A**GATATC**GCCTCGTACAGCTCACCGG | ΔentB |
|  | RICENT/B | BamHI/NheI | AA**GGATCC**A**GCTAGC**GGGTGCTCACCACCGACG |  |
|  | RICENT/C | PstI/NheI | ATAT**CTGCAGCTAGC**GGTTCACGGGCAGTTCG |  |
|  | RICENT/D | SpeI | A**ACTAGT**GCTCGCACCGTTCTGCACG |  |
| pISENT-AtBh | RISENT/A | EcoRV | A**GATATC**CACTTGGTCAGGACGATGTCGC | ΔentC |
|  | RISENT/B | NdeI/NheI | ATAATT**CATATGCTAGC**GTGGTGGCCCAGTCCTC |  |
|  | RISENT/C | PstI/NheI | ATAT**CTGCAG**T**GCTAGC**TGCCGAGCAGGGTGTG |  |
|  | RISENT/D | SpeI | A**ACTAGT**CGGGAGAACTCACCGAGGACACC |  |

[a] Restriction site in bold and underlined in primer sequence.

**Table S2.** Primers used in this work for the amplification of DNA regions used in gene expression experiments.

| **Genes from caboxamycin producer *Streptomyces* sp. NTK937** | | | | |
| --- | --- | --- | --- | --- |
| **Construct** | **Primer** | **Nucleotide sequence (5’ 🡪 3’)** | **Gene** | **Description^[a]^** |
| pT-cbxR | SARP/fw | A**GAATTC**GCCGACGCCACGCTGTAGC | *cbxR* | EcoRI |
|  | SARP/rv | TT**GGATCC**GGGCGATGTCGTCCTCC |  | BamHI |
| pT-cbxA | SS/fw | AA**GGATCC**GATCTGCGACGTGTCGCCGTC | *cbxA* | BamHI |
|  | SS/rv | A**GAATTC**GGACTCGATCCCGGACTCCTC |  | EcoRI |
| pT-cbxB | OA/fw | AA**GGATCC**GAGAAGCTGGGCAGCGTCG | *cbxB* | BamHI |
|  | OA/rv | A**GAATTC**GACAGCAGGTCCGCGAGGTTCTC |  | EcoRI |
| pHT-cbxC | ASL/fw | AA**AGATCT**CAACACCTGTGGCGGACCG | *cbxC* | BglII |
|  | ASL/rv | A**GAATTC**GCTGTCCTGTGCTGTGGTG |  | EcoRI |
| pT-cbxD | ACP/fw | AA**AGATCT**GGCCTCGGCAAGGTCGACAAG | *cbxD* | BglII |
|  | ACP/rv | A**GAATTC**GTCCGCCGTGCAGAGGTG |  | EcoRI |
| pT-cbxE | AH/fw | AA**AGATCT**GGAGTGAAGGTCTCCTTCCGC | *cbxE* | BglII |
|  | AH/rv | A**GAATTC**GGTGTTGTCGGGCATGGTG |  | EcoRI |
| pKT-cbxF | DAHP/fw | AA**GGATCC**GTCGCCGAGAAGGACCTGAC | *cbxF* | BamHI |
|  | DAHP/rv | A**GAATTC**CGAGGTCAGCTCCTTGGC |  | EcoRI |
| pKT-cbxG | 23DH/fw | AA**GGATCC**GCCTACACGACGCTCTGC | *cbxG* | BamHI |
|  | 23DH/rv | A**GAATTC**GCAGATCGCCCTGGGTGG |  | EcoRI |
| pKT-cbxH | IC/fw | AA**AGATCT**GTTCCTGCTGTCCGACCAGG | *cbxH* | BglII |
|  | IC/rv | A**GAATTC**TGCCCACCATCAGTTCGAGCC |  | EcoRI |
| pHT-cbxI | AS/fw | TT**AGATCT**GGAGTACGTCGCCACCC | *cbxI* | BglII |
|  | AS/rv | A**GAATTC**GTCAAGGCGGTCCAGACGC |  | EcoRI |
| pT-cbxA’ | SSALT/fw | AA**GGATCC**GAAGGCACCACACGATGAGCACC | *cbxA’* | BamHI |
|  | SSALT/rv | A**GAATTC**ATGGACCGATGGTCTCACACGAAC |  | EcoRI |
| pHT-cbxC’ | ASLALT/fw | TT**GGATCC**GTGACGGACTGGCGTGCTTC | *cbxC’* | BamHI |
|  | ASLALT/rv | A**CAATTG**GCAGATGGACGGAGTTGGC |  | MunI |
| pHT-cbx[FGHI]’ | ASALT/fw | A**GAATTC**GATGGTCGTCTGTGCTCAGCG | *cbx*  *[FGHI]’* | EcoRI |
|  | DAHPALT/rv | TT**AGATCT**CGGTGAACCGCATGTGCG |  | BglII |
| pHT-orf-7 | ASLm7/fw | AA**GGATCC**GGTACAGCCGTGGATCACCGACC | *orf-7* | BamHI |
|  | ASLm7/rv | A**GAATTC**GATCTTGGGTGTGGTGAGGCTGC |  | EcoRI |
| pKT-entA | DHENT/fw | A**GAATTC**GACACTCGGAGAGGCGGACATG | *entA* | EcoRI |
|  | DHENT/rv | AA**GGATCC**GAAGGCCGTGGTCAAGTGAGC |  | BamHI |
| pKT-entB | ICENT/fw | A**GAATTC**GGAGCCGTGAGGTCGCTGG | *entB* | EcoRI |
|  | ICENT/rv | AA**GGATCC**GCACCGAAAGGCCCTCCCTC |  | BamHI |
| **Genes from nataxazole producer *Streptomyces* sp. Tü6176** | | | |  |
| pT-natR4 | SAR29fw | AAG**AGATCT**GCGTGCGGTAGAGATTCTG | *natR4* | BglII |
|  | SAR29rv | AAT**GAATTC**GGAGGCGGGGTAATACTGA |  | EcoRI |
| pT-cf_20720 | SSTUfw | AA**GGATCC**TCCTTCTGGGAGCGGCG | CF54_20720 | BamHI |
|  | SSTUrv | A**GAATTC**CTCGACCGTGGATCTCGGC |  | EcoRI |
| pT-natS | ACPsintfw | AAA**GAATTC**CGCCAGATGCCCTGCTCG | *natS* | EcoRI |
|  | ACPsintrv | AAA**CAATTG**CCCCCACCTCTTCCCCCA |  | MunI |
| pHT-natL1 | Tu-ASL21/fw | A**GAATTC**GTTCGTCTTCGGTCGGGAATGCG | *natL1* | EcoRI |
|  | Tu-ASL21/rv | AA**GGATCC**GCGACCATGTCCTGCTGATCGG |  | BamHI |
| pHT-natL2 | Tu-ASL24/fw | AA**GGATCC**GAGCACAGGACAGCACCGGG | *natL2* | BamHI |
|  | Tu-ASL24/rv | A**GAATTC**GGCGTCAGAAGTACATGACCGC |  | EcoRI |
| pT-natAC1 | Tu-ACP20/fw | A**GAATTC**GACCTTGTCGTCCACGGTCAG | *natAC1* | EcoRI |
|  | Tu-ACP20/rv | AA**GGATCC**TCGCCGACCTCAAGCAG |  | BamHI |
| pT-natAC2 | ACP1530/fw | AAA**CAATTG**AAAAGCCGTTCGGAGAGGAT | *natAC2* | MunI |
|  | ACP1530/rv | AAA**GAATTC**AGGAGGAGCCGGGGAAG |  | EcoRI |
| pT-natAM | 1519/fw | AAG**GAATTC**TAGACCGTTCACCAGGTAGGCG | *natAM* | EcoRI |
|  | 1519/rv | AAT**CAATTG**CCCACTTCGACTCGCTGC |  | MunI |
| pKT-natDB | 1518/fw | t**atggat**cc**tctaga**cggacctcggggaagtcgg | *natDB* | ClaI/XbaI |
|  | 1518/rv | taa**agatctactagt**cgaccgctacctccaggcact |  | BglII/SpeI |
| pKT-natIS | ISCOMP1 | AGT**AGATCT**CACCACCGGTTCACCCCC | *natIS* | BglII |
|  | ISCOMP2 | ATG**CAATTG**AACCTAGGGGCCAGTTCCGTCAGGGAC |  | MunI |
| pHT-natAN | ASEXP-1 | aa**agatct**cgtgggaggcccctcatgaca | *natAN* | BglII |
|  | ASEXP-2 | a**gaattc**agtcacgccgtcagctccgct |  | EcoRI |
| pT-natR2 | TetR14/fw | ATG**AGATCT**GCATGGTCGGTTCTGACA | *natR2* | BglII |
|  | TetR14/rv | ATG**GAATTC**GGCACTTCAGCCTGTTCC |  | EcoRI |
| pT-natR3 | TetR27/fw | TAA**GAATTC**CGTTCGGCTGGAGGTACA | *natR3* | EcoRI |
|  | TetR27/rv | TGA**GGATCC**AGTGTTGACCATGCCTTGC |  | BamHI |

[a] Restriction site in bold in primer sequence.

**Table S3.** Primers used in this work for the amplification of resistance genes and heterologous expression of caboxamycin biosynthesis gene cluster

| **Primer** | **Nucleotide sequence (5’ 🡪 3’)** | **Description** |
| --- | --- | --- |
| Tsr/fw | AG**TCTAGA**AATACTTCATATGCGGGGATC | Amplification of *tsr* (thiostrepton resistance gene) from pSETeTc, with XbaI sites |
| Tsr/rv | TA**TCTAGA**AACAGAGGCGCTTATCGG |
| Kan/fw | TATA**TCTAGA**TAGACTGGGCGGTTTTATGG | Amplification of *aph*(3)II (kanamycin resistance gene) from pCAP01, with XbaI sites |
| Kan/rv | TATA**TCTAGA**GAAATCTCGTGATGGCAGGT |
| CABTAR/A | TATA**ACTAGT**ctgctcaccgagtacctgaa | Amplification of the “arms” used for constructing pCABTAR for heterologous expression of the structural genes of caboxamycin biosynthesis |
| CABTAR/B | TATA**GGATCC**gaccatcgcgaagacgag |
| CABTAR/C | TATA**GGATCC**ccgtacctcaaggagatgga |
| CABTAR/D | TATA**CTCGAG**tgtagaagccgacgagttcc |

**Figure S1.** UPLC analysis showing production of salicylic acid (**4**) in cultures of *S. albus* J1074 carrying pEM4T, pT-SS (*cbxA*), pT-SSALT (*cbxA’*) or pT-SSTu (CF54_20720), grown in R5A medium for 7 days. UPLC chromatograms were analyzed at 234 nm. Asterisks point to paulomycins and paulomenols produced by *S. albus* J1074. Salicylate synthase CF54_20720 is from nataxazole producer *Streptomyces* sp. Tü6176.

**Figure S2.** UPLC analysis of *Streptomyces* sp. NTK937 wild-type (WT) and mutant strains. Each mutant strain was generated by individual inactivation of the indicated *orfs* at the boundaries of the caboxamycin biosynthesis cluster. Extracts were obtained from cultures grown in R5A medium for 7 days. UPLC chromatograms were analyzed at 330 nm. Labeled peaks correspond to: caboxamycin A (**1**) and *O*-methylcaboxamycin (**2**).

**Figure S3.** A) Genetic complementation of ΔcbxR mutant strain**.** B) Ectopic expression of *cbxR* and nataxazole biosynthesis pathway regulatory genes *natR4*, *natR2* and *natR3* in *Streptomyces* sp. NTK3987 (WT). Each plasmid is expressing the appropriate intact gene(s) under the control of *ermE*p*. Extracts were obtained from cultures grown in R5A medium for 7 days. UPLC chromatograms were analyzed at 330 nm. Labeled peaks correspond to: caboxamycin A (**1**) and *O*-methylcaboxamycin (**2**).

**Figure S4.** Genetic complementation of ΔcbxA mutant strain**.** Each plasmid is expressing the appropriate intact gene(s) under the control of *ermE*p*. Extracts were obtained from cultures grown in R5A medium for 7 days. UPLC chromatograms were analyzed at 330 nm. Labeled peaks correspond to: caboxamycin A (**1**), *O*-methylcaboxamycin (**2**) and 3’-hydroxycaboxamycin (**3**).

**Figure S5.** Genetic complementation of ΔcbxB mutant strain**.** Each plasmid is expressing the appropriate intact gene(s) under the control of *ermE*p*. Extracts were obtained from cultures grown in R5A medium for 7 days. UPLC chromatograms were analyzed at 330 nm. Labeled peaks correspond to: caboxamycin A (**1**) and *O*-methylcaboxamycin (**2**).

**Figure S6.** Genetic complementation of ΔcbxC/C’ mutant strain**.** Each plasmid is expressing the appropriate intact gene(s) under the control of *ermE*p*. Extracts were obtained from cultures grown in R5A medium for 7 days. UPLC chromatograms were analyzed at 330 nm. Labeled peaks correspond to: caboxamycin A (**1**), *O*-methylcaboxamycin (**2**) and 3’-hydroxycaboxamycin (**3**).

**Figure S7.** Genetic complementation of ΔcbxD mutant strain**.** Each plasmid is expressing the appropriate intact gene(s) under the control of *ermE*p*. Extracts were obtained from cultures grown in R5A medium for 7 days. UPLC chromatograms were analyzed at 330 nm. Labeled peaks correspond to: caboxamycin A (**1**) and *O*-methylcaboxamycin (**2**).

**Figure S8.** Genetic complementation of ΔcbxE mutant strain**.** Each plasmid is expressing the appropriate intact gene(s) under the control of *ermE*p*. Extracts were obtained from cultures grown in R5A medium for 7 days. UPLC chromatograms were analyzed at 330 nm. Labeled peak correspond to *O*-methylcaboxamycin (**2**).


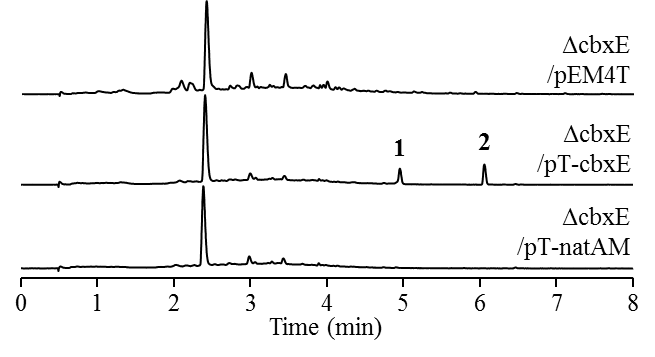


**Figure S9.** Genetic complementation of ΔcbxG/[FGHI]’/entA mutant strain**.** Each plasmid is expressing the appropriate intact gene(s) under the control of *ermE*p*. Extracts were obtained from cultures grown in R5A medium for 7 days. UPLC chromatograms were analyzed at 330 nm. Labeled peaks correspond to: caboxamycin A (**1**) and *O*-methylcaboxamycin (**2**).

**Figure S10.** Genetic complementation of ΔcbxH/[FGHI]’/entB mutant strain**.** Each plasmid is expressing the appropriate intact gene(s) under the control of *ermE*p*. Extracts were obtained from cultures grown in R5A medium for 7 days. UPLC chromatograms were analyzed at 330 nm. Labeled peaks correspond to: caboxamycin A (**1**) and *O*-methylcaboxamycin (**2**).

**Figure S11.** Genetic complementation of ΔcbxI/[FGHI]’ mutant strain**.** Each plasmid is expressing the appropriate intact gene(s) under the control of *ermE*p*. Extracts were obtained from cultures grown in R5A medium for 7 days. UPLC chromatograms were analyzed at 330 nm. Labeled peaks correspond to: caboxamycin A (**1**) and *O*-methylcaboxamycin (**2**).

**Figure S12.** A) UPLC analysis of Streptomyces sp. NTK937 wild type strain (WT) and DAHP synthase–deficient mutant strains. B) UPLC analysis of *Streptomyces* sp. NTK937 wild type strain (WT) and DAHP synthase-deficient mutant strains in cultures supplemented with each individual aromatic aminoacid or a mixture of all three. Extracts were obtained from cultures grown in R5A medium for 7 days. UPLC chromatograms were analyzed at 330 nm. Labeled peaks correspond to: caboxamycin A (**1**) and *O*-methylcaboxamycin (**2).**


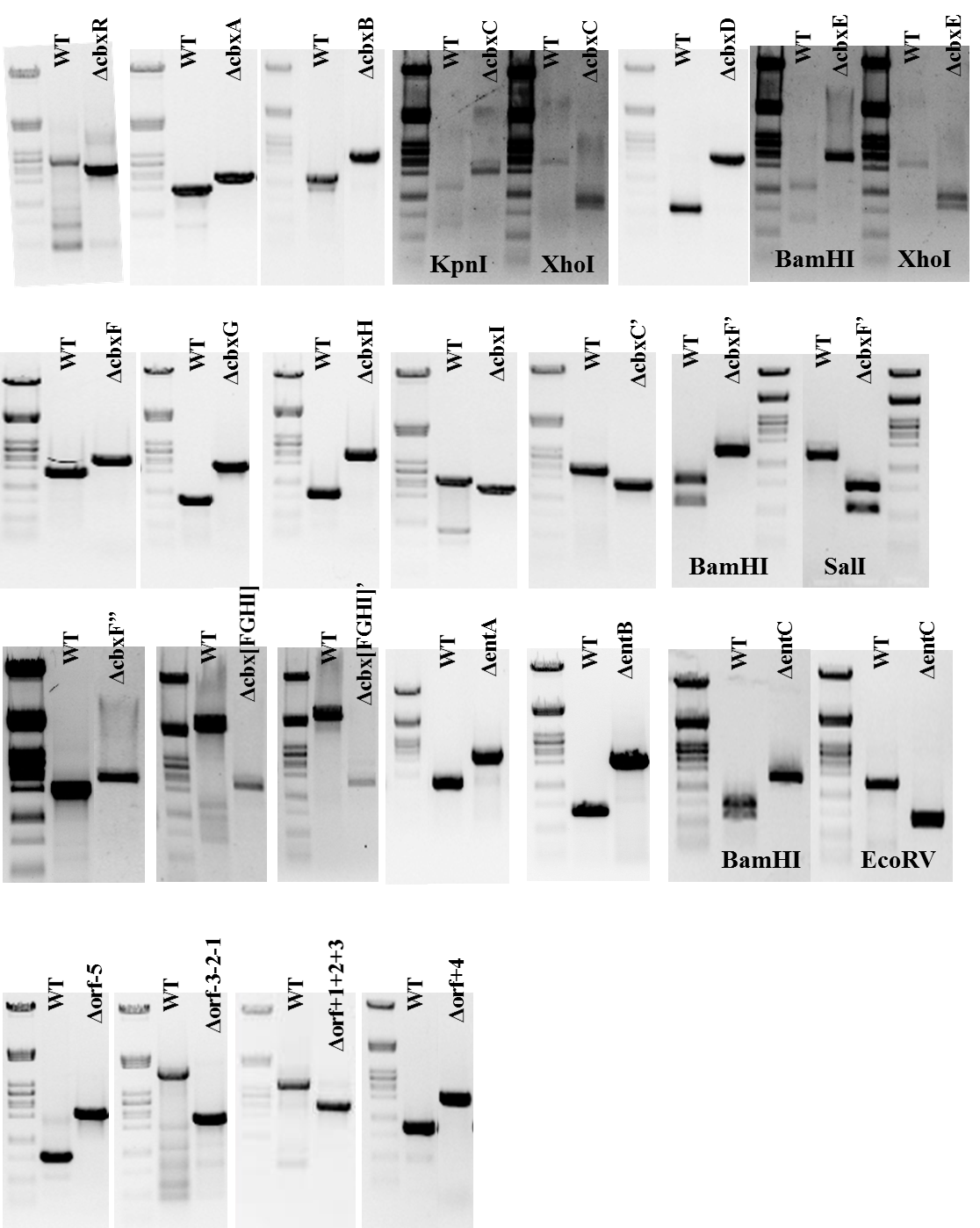
**Figure S13.** PCR analysis of *Streptomyces* sp. NTK 937 mutant strains. Chromosomal DNA from each mutant was used to verify the correct location of the resistance marker. The oligoprimers used are listed at Table S2 and were designed to amplify the entire corresponding gene. Fragments differing less than 100 bp between wild-type and mutant strains were subjected to differential restriction enzyme digestion Lambda DNA digested with *Pst*I has been used as molecular-weight size marker. All PCR products generated were sequenced.

**Figure S14.** ^1^H NMR spectrum of *O*-methylcaboxamycin (**2**) (DMSO-*d_6_*, 500 MHz, 24 °C).

**Table S4.** *O*-methylcaboxamycin (**2**) ^13^C and ^1^H NMR data acquired in DMSO-*d_6_* (500 MHz, 24 °C)

| **Position** | **δ ^13^C (ppm)^[a]^** | **δ ^1^H (ppm)** | ***J* (Hz)** |
| --- | --- | --- | --- |
| 1’ | n. d. | - |  |
| 2’ | n. d. | - |  |
| 3’ | 117.8 | 7.17, d | 7.9 |
| 4’ | 134.8 | 7.58, t | 7.8 |
| 5’ | 120.2 | 7.12, t | 7.8 |
| 6’ | 127.9 | 8.06, d | 7.8 |
| 2 | n. d. | - |  |
| 3a | n. d. | - |  |
| 4 | n. d. | - |  |
| 5 | 127.3 | 8.02, d | 7.8 |
| 6 | 125.6 | 7.61, t | 8 |
| 7 | 116.1 | 8.16, d | 8 |
| 7a | n. d. | - |  |
| 8 | n. d. | - |  |
| COOMe | 52.8 | 3.98, s |  |

                 [a] δ^13^C determined from the indirect dimension of HSQC

spectrum. (n. d. = not determined)

**Figure S15**. ^1^H NMR spectrum of 3’-hydroxycaboxamycin (**3**) (DMSO-*d_6_*, 500 MHz, 24 °C).

**Figure S16**. Expansion of the aromatic region in the proton spectrum of 3’-hydroxycaboxamycin (**3**).

**Table S5.** 3’-hydroxycaboxamycin (**3**) ^13^C and ^1^H NMR data acquired in DMSO-*d_6_* (500 MHz, 24 °C)

| **Position** | **δ ^13^C (ppm)^[a]^** | **δ ^1^H (ppm)** | ***J* (Hz)** |
| --- | --- | --- | --- |
| 1’ | 110.2 | - |  |
| 2’ | 148.0 | - |  |
| 3’ | 146.7 | - |  |
| 4’ | 120.3 | 7.06, d | 8.0 |
| 5’ | 120.3 | 6.93, dd | 8.0, 8.0 |
| 6’ | 117.5 | 7.50, d | 8.0 |
| 2 | 164.4 | - |  |
| 3a | 139.1 | - |  |
| 4 | 122.0 | - |  |
| 5 | 127.6 | 8.00, d | 8.1 |
| 6 | 125.8 | 7.58, dd | 8.1, 8.1 |
| 7 | 115.8 | 8.12, d | 8.1 |
| 7a | 149.9 | - |  |
| 8 | 166.0 | - |  |
| OH (2’) | - | 11.9, s |  |
| OH (3’) | - | 9.51, s |  |
| COOH | - | 13.3, br s |  |

                 [a] δ^13^C determined from HQSC and HMBC spectra
